# Supplementary material for: Reduced ultrafine particle levels in São Paulo’s atmosphere during shifts from gasoline to ethanol use
Source: Nat Commun. 2017 Jul 18;8:77. doi: 10.1038/s41467-017-00041-5 (PMC5516031; doi:10.1038/s41467-017-00041-5)
Supplement: Supplementary file 1 — Supplementary Information [file 41467_2017_41_MOESM1_ESM.pdf]

File name: Supplementary Information
Description: Supplementary Figures, Supplementary Tables, Supplementary Notes and Supplementary References

File name: Peer Review File
Description:

Supplementary Figure 1. Sampling sites.

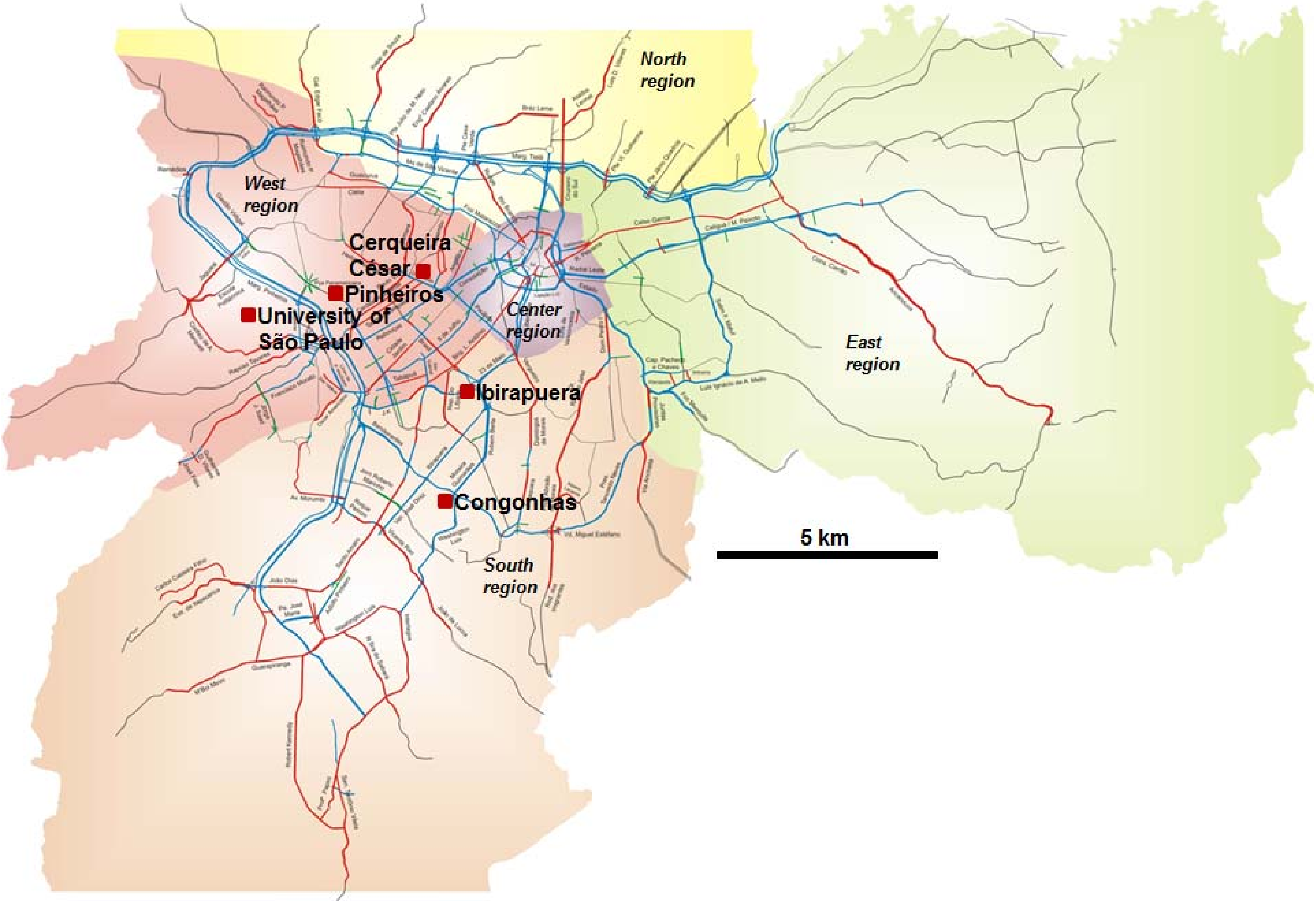

The figure indicates the location of sampling for all three datasets: Cerqueira César, Ibirapuera and Pinheiros (24-hour filter); Congonhas, Pinheiros and University of São Paulo/IPEN (beta continuous); University of São Paulo (DMPS, CPC, MAAP).

Supplementary Figure 2. Harmonization between DMPS and CPC.

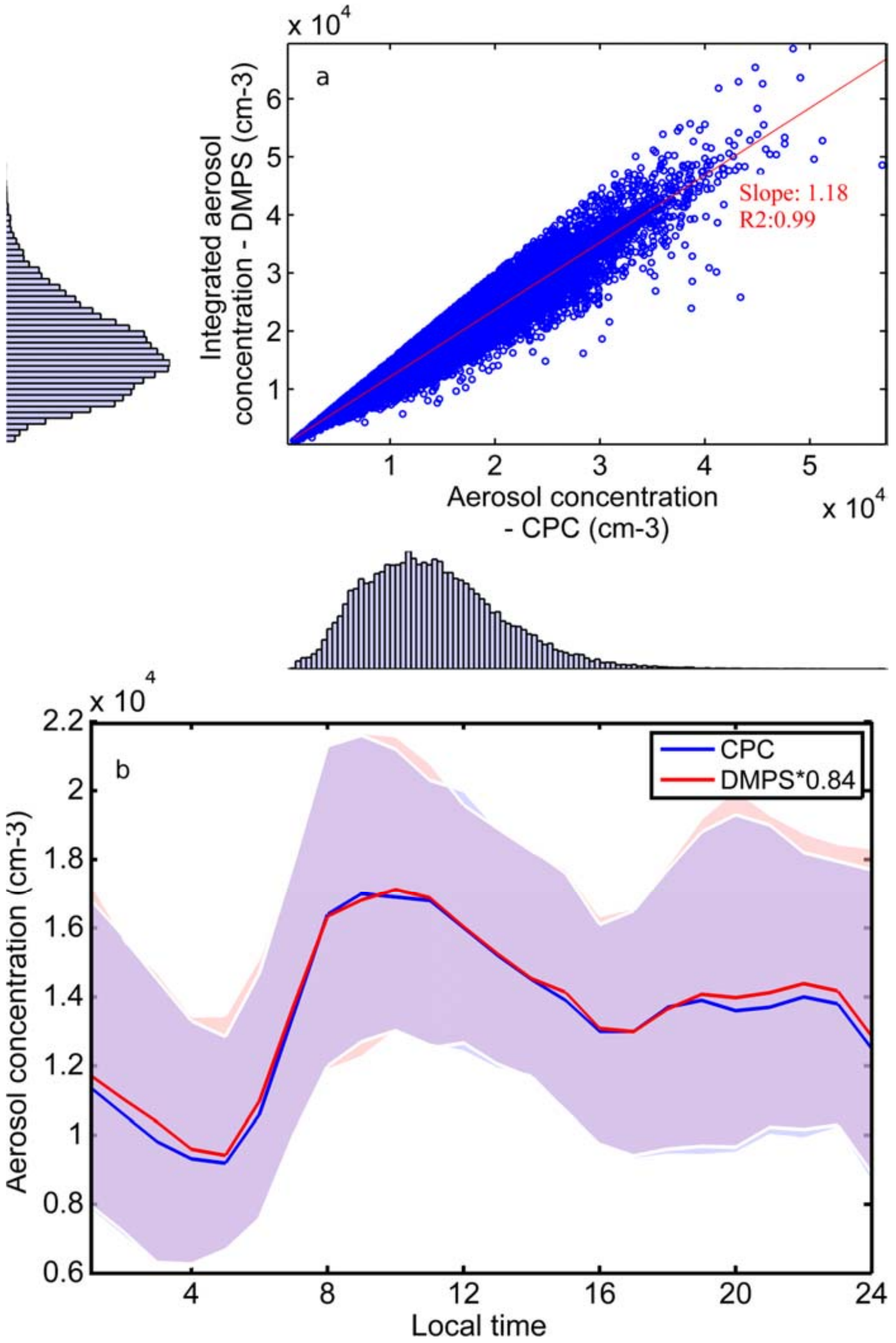

The panels compare aerosol number concentrations integrated from the DMPS and from an independently operated CPC. (**a**) Scatterplot and line of best fit between values in both datasets. (**b**) Diurnal variation (median, marked by the thick lines) and variability (the interquartile range is shaded) in each dataset.

Supplementary Figure 3. Variation in PM2.5 and BC mass concentrations across sites and methods.

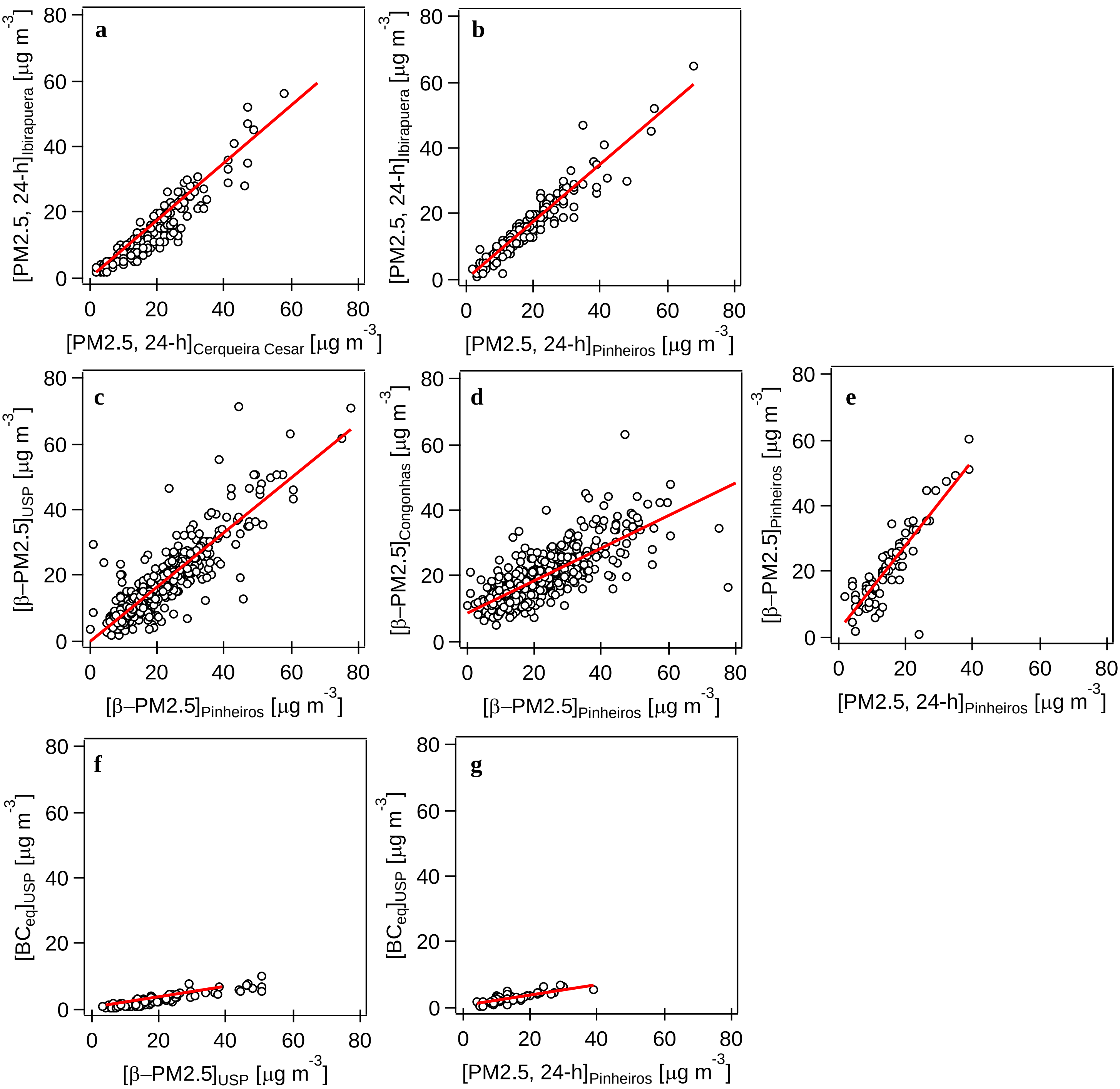

The panels compare mass concentrations, in μg m^{-3}, measured on the same dates across the different sampling sites and methods. An observation in the scatterplots is a date. In each

panel, the red line shows the best linear predictor (line of best fit), not the 45-degree line. Sources: CETESB, own measurements. (**a**) Comparison across sampling sites: Ibirapuera against Cerqueira César. 24-hour filter measurements of PM2.5 mass concentration. (**b**) Comparison across sampling sites: Ibirapuera against Pinheiros. 24-hour filter measurements of PM2.5 mass concentration. (**c**) Comparison across sampling sites: University of São Paulo against Pinheiros. Beta-continuous measurements of PM2.5 mass concentration (hourly aggregated to 24-hour mean). (**d**) Comparison across sampling sites: Congonhas against Pinheiros. Beta-continuous measurements of PM2.5 mass concentration (hourly aggregated to 24-hour mean). (**e**) Comparison across methods: Beta-continuous against 24-hour filter measurements. Both measurements of PM2.5 mass concentration at the Pinheiros site (24-hour mean). (**f**) Comparison across parameters (and sampling sites): MAAP measurements of black carbon mass concentration against beta-continuous measurements of PM2.5 mass concentration at nearby sites in the University of São Paulo campus (hourly measures aggregated to 24-hour mean). (**g**) Comparison across parameters (and sampling sites): MAAP measurements of black carbon mass concentration at the University of São Paulo site against 24-hour filter measurements of PM2.5 mass concentration at the Pinheiros site across the Pinheiros river (hourly measures aggregated to 24-hour mean).

Supplementary Figure 4. Shifting gasoline-ethanol fuel mix and relative prices between November 2008 and May 2013.

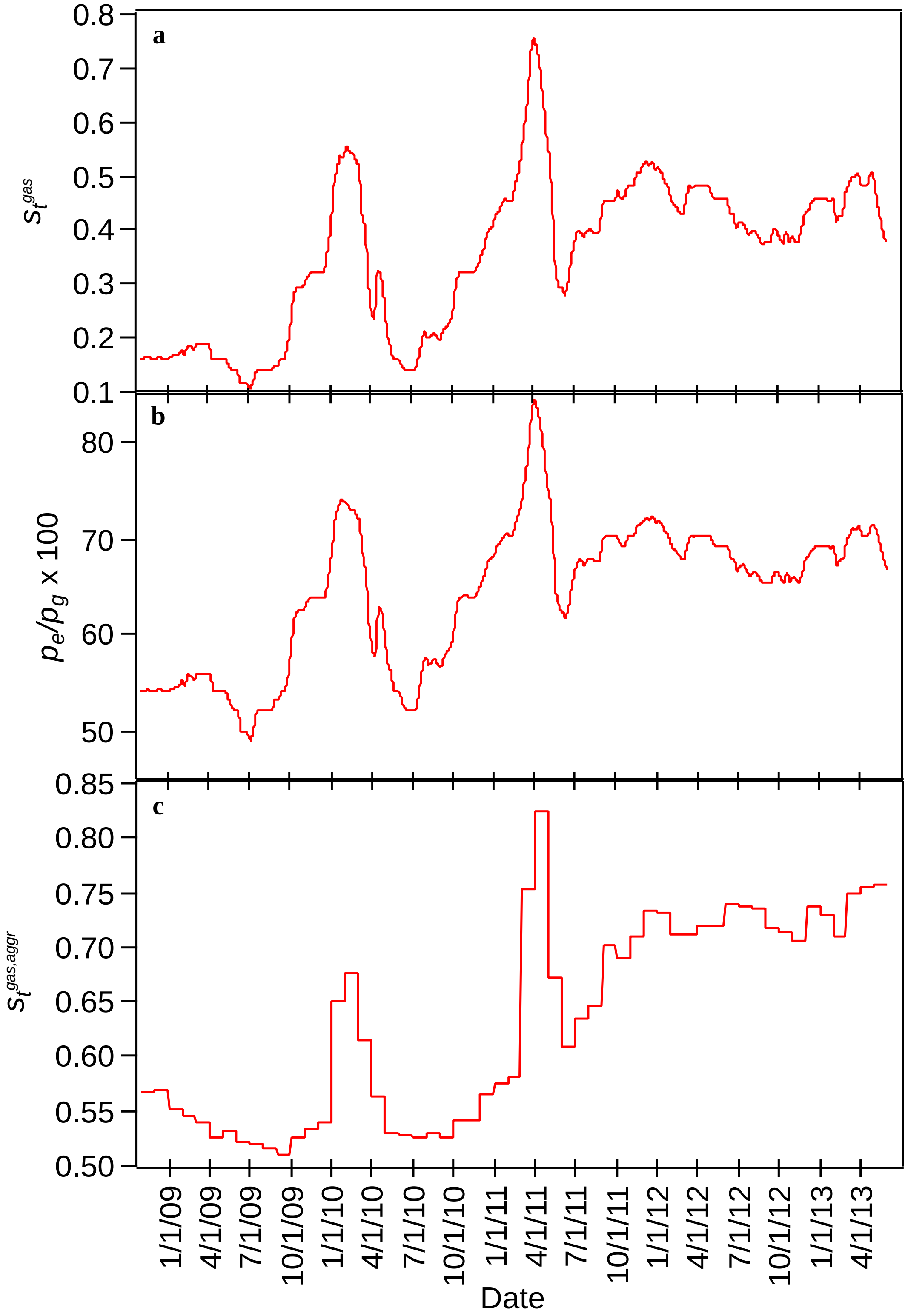

We report temporal variation over the sampling period for the most extended particle dataset. Sources: authors' estimates, ANP. (**a**) The gasoline share in the flex-fuel light-vehicle fleet, denoted by $\hat{s}_t^{\text{gas}}$, generated from a first-step consumer demand model[1]. This indicates the proportion of flex-fuel vehicles burning blended gasoline (E20 or E25). The proportion of flex-fuel vehicles burning ethanol (E100) is one minus the gasoline share. (**b**) The consumer ethanol-to-gasoline price ratio (median ratio of regular-grade prices per liter in large weekly samples of São Paulo city retailers). (**c**) The blended gasoline share in the aggregate fleet of engines that are powered by either blended gasoline (E20 or E25) or ethanol (E100), for the state of São Paulo, denoted by $s_t^{\text{gas,aggr}}$. We calculate this share from monthly fuel quantities for blended gasoline and hydrated ethanol reported by wholesalers. To compute $s_t^{\text{gas,aggr}}$ we adjust for differences in energy content by converting quantities by fuel from cubic meters to light-vehicle distance traveled, given assumptions on the fleet's fuel economy. Shares based on fuel quantities expressed in energy-adjusted barrels of oil equivalent, reported by the same data provider (ANP) in addition to cubic meters, are similar.

Supplementary Figure 5. Monthly price indices for diesel oil and light-vehicle fuels in the São Paulo metropolis from October 2008 to May 2013.

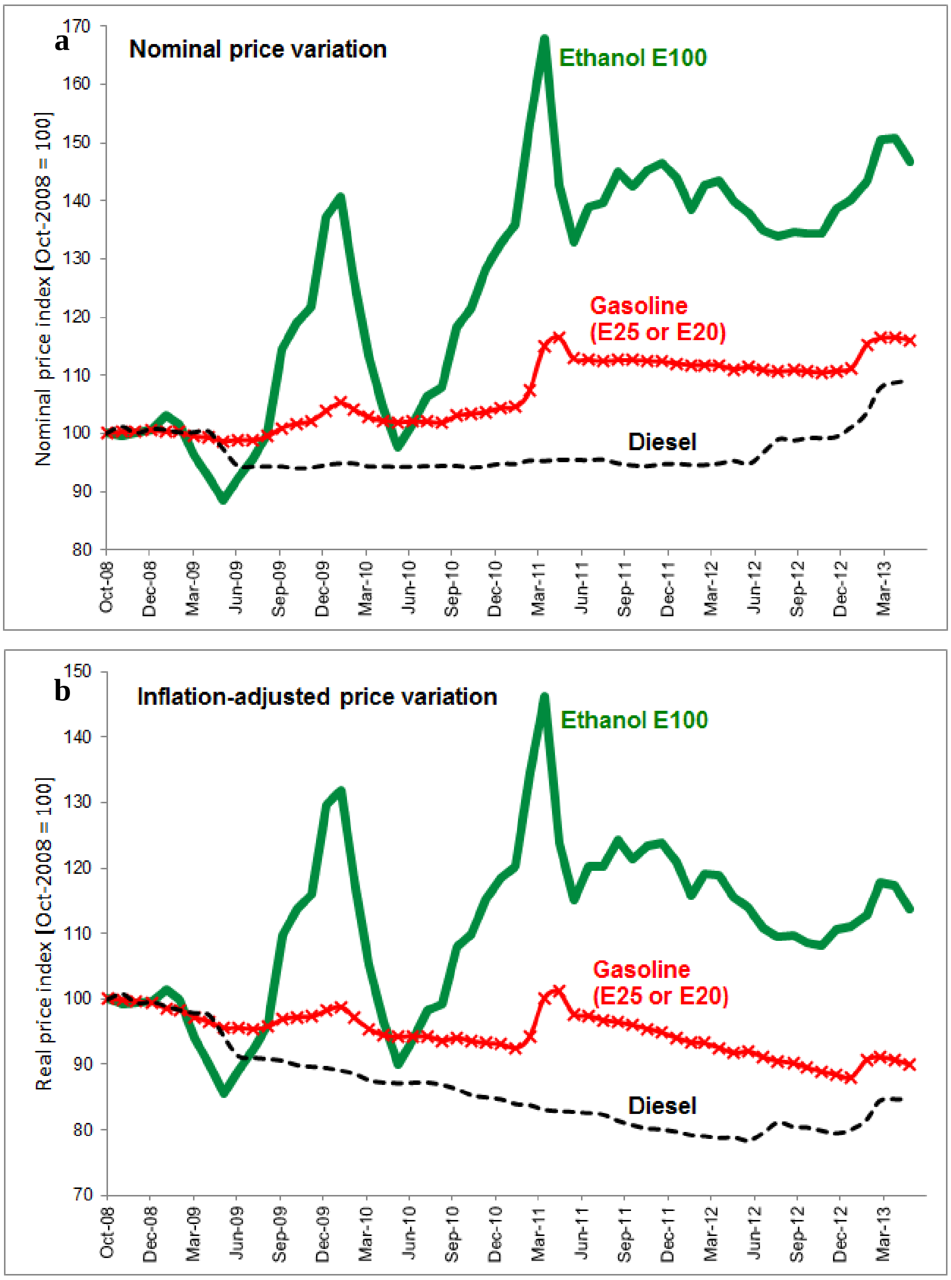

Indices for price at the pump for diesel (dashed black line), regular-grade gasoline (E25 or E20, crossed red line), and regular-grade ethanol (E100, thick green line), (**a**) not adjusted for inflation, and (**b**) deflated to account for variation in the economy-wide price level (we use the IPCA Brasil, a Consumer Price Index, to do this). Base October 2008 = 100. Source: IBGE (Brazilian Institute for Geography and Statistics).

Supplementary Figure 6. Ridership on diesel buses in the public transport system in the São Paulo metropolis from November 2008 to May 2013.

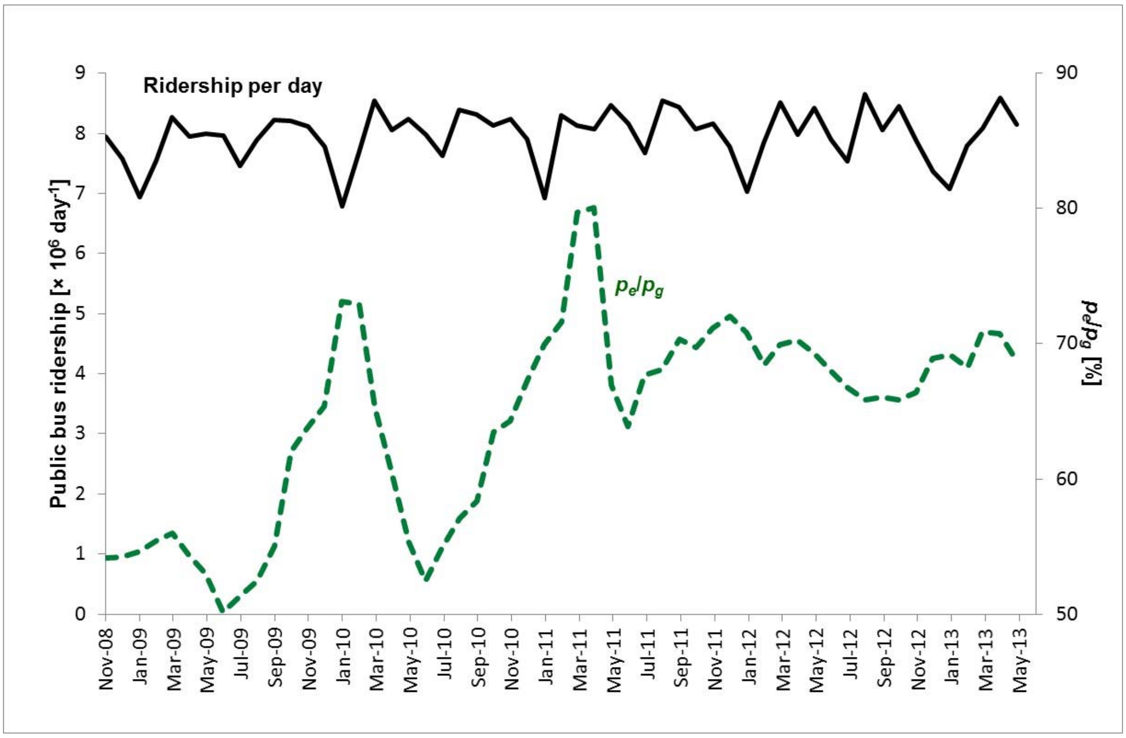

Average daily rates of ridership in millions of passengers per day are reported by dividing the month's total ridership by the number of calendar days in the month (left vertical axis). Average ethanol-to-gasoline price ratio across the city's pumps (right vertical axis). Sources: SPTrans (Prefeitura de São Paulo, Transportes), ANP.

Supplementary Figure 7. Frequency of diesel buses passing through the Armando Salles de Oliveira campus of the University of São Paulo.

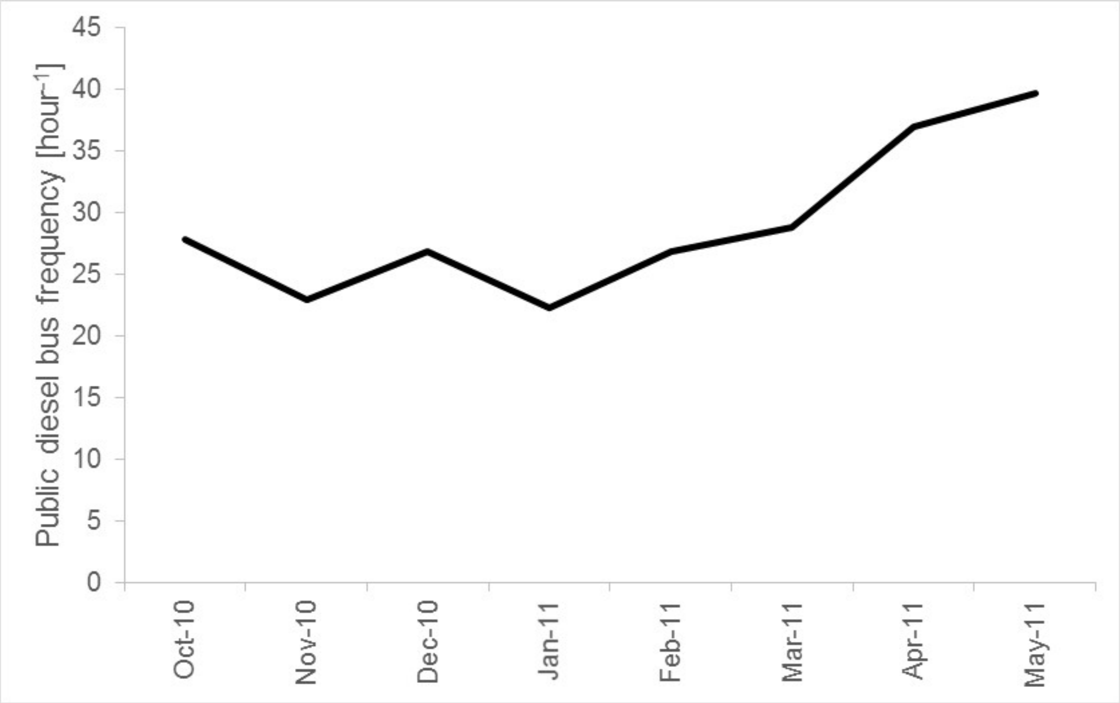

Actual frequency of public transit diesel buses (in vehicles hour^{-1}) passing, during the weekday morning commute hour from 09:00 to 09:59, in either direction within a ground-level distance of 400 m from the four-storey building on whose rooftop the DMPS/CPC instrumentation was deployed. The bus lines include 177H-10, 701U-10, 702U-10, 7181-10, and 8012-10. Bus line 8012-10 was added on March 29, 2011. The number of vehicles transiting in either direction is summed across weekdays in each month of the submicron particle sample, between October 2010 and May 2011 (see Table 4 in the main text), and divided by the number of weekdays in that month. The submicron particle sampling site was chosen due to limited influence of local sources. Source: SPTrans (Prefeitura de São Paulo, Transportes, InfoTrans "Desempenho Operacional – Bilhetagem").

Supplementary Figure 8. Monthly wholesale diesel fuel shipments for the state of São Paulo over the first semester of 2011 compared to the first semester in other years.

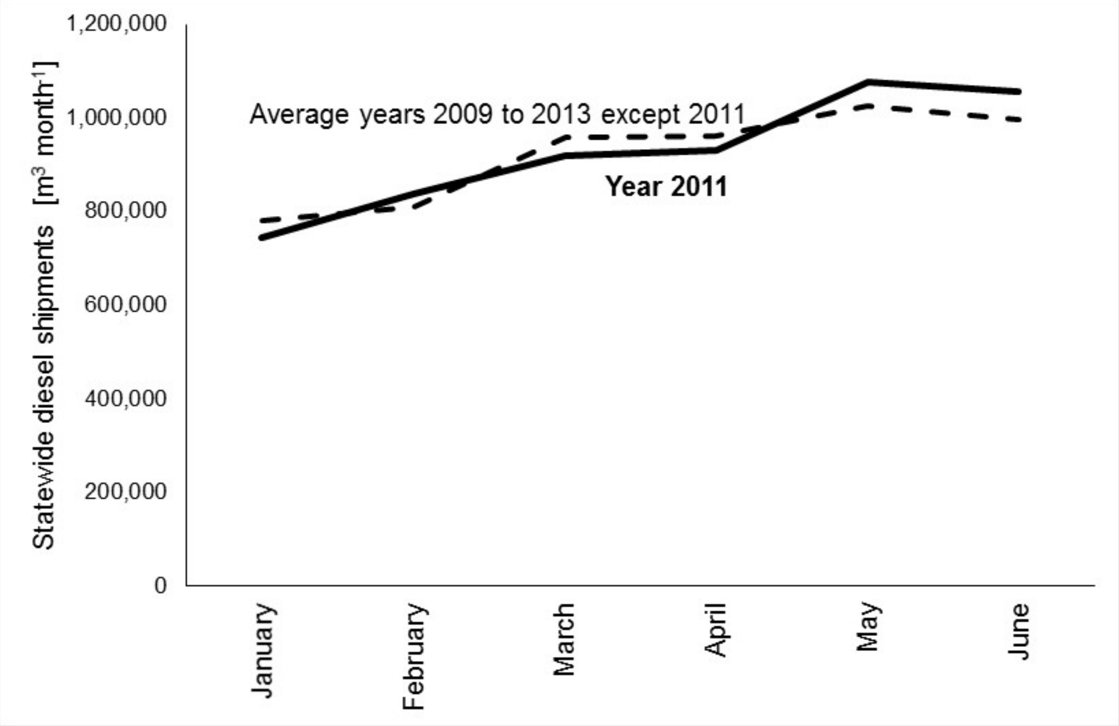

Shipments include the state-wide highway market. During semester 1, 2011, diesel prices hardly varied whereas ethanol prices fluctuated markedly relative to gasoline prices (Supplementary Fig. 5). Source: ANP.

Supplementary Note 1. PM2.5 concentrations based on 24-hour filter measurements. Supplementary Tables 1 and 2 provide sensitivity analysis with regard to the estimates reported in Table 2, column (2) in the main text, where the dependent variable is the 24-hour PM2.5 mass concentration. Supplementary Table 1 reports on second-step particle regression models in which we progressively add more controls, and where the gasoline share $\hat{s}_t^{gas}$ is imputed from a first-step estimated demand model and a bootstrap procedure corrects for sampling variation in the first step. Supplementary Table 2 reports on particle regression models with sufficiently rich controls, and where we conduct sensitivity analysis by varying the gasoline share specification, namely, using an instrumental variable in a first-stage regression, or basing it on available aggregate wholesaler data, as described in Methods.

The bottom row of Supplementary Table 1 reports a mean 24-hour PM2.5 mass concentration of 13.8 μg m^{-3} in the sample of 511 site-date observations (3 sites, every 6 days, 2008 to 2013, excluding the colder months of June to September). This mean exceeds the US EPA annual PM2.5 standard of 12 μg m^{-3} [2].

In column (1), absent controls, PM2.5 mass concentration is not significantly associated with the gasoline share. To see this, notice that the point estimate on the gasoline share, -1.2 μg m^{-3} (for a 30% to 80% shift in the share of the flex fleet) is well within 1.96 standard errors (1.96×1.8=3.5 μg m^{-3}) of 0 μg m^{-3}. (All Supplementary Tables 1 to 8 report estimated standard errors (s.e.) inside parentheses; the 95% confidence interval is then given by: point estimate ± 1.96×standard error.) This conclusion does not change as we gradually increase the number of controls and thus the explanatory power (R^2) of the regression model. In column (2), we add site-specific time trends, as well as fixed effects (i.e., allow different intercepts) for every week of the year (e.g., week 50) and for every day of the week and type of day (e.g., Mondays that were not public holidays). Point estimates for drifts at Cerqueira César, Pinheiros and Ibirapuera are +1.0, +0.4 and -1.0 μg m^{-3} $year^{-1}$, respectively (not shown for brevity).

As we add controls, the zero (or, more accurately, the not-significantly-different-than-zero) effect of the gasoline share survives, and is estimated with increased precision, i.e., standard errors fall. Introducing information on (mean daily) contemporaneous meteorological and atmospheric conditions, and to a lesser extent citywide and local road

traffic conditions, adds substantial explanatory power to the model. The R^2 in column (3) rises to 74% compared to 29% in column (2). This is important, since all particle regression models that we estimate in this work, even those based on samples shorter than one year, control for meteorological, atmospheric and traffic conditions. On top of controlling for systematic seasonal variation and excluding the colder months of June to September, PM2.5 concentrations increase in (mean) temperature (i.e., +1.3 μg m^{-3} per +1 °C, with s.e. 0.2 μg m^{-3}), decrease in wind speed (-5.8 μg m^{-3} per +1 m s^{-1}, with s.e. 1.1 μg m^{-3}), and tend to be higher when thermal inversions are recorded. (For brevity, the latter effects are not shown; we describe these below.) In column (3), raising the penetration of blended gasoline in the flex-fuel light-vehicle fleet from 30% to 80% (equivalently, lowering the ethanol share from 70% to 20%) is associated with a small and statistically insignificant 0.1 μg m^{-3} increase in the PM2.5 mass concentration, with a s.e. of 2.0 μg m^{-3}.

The remaining columns in Supplementary Table 1 report variations around the specification in column (3). These point to the robustness of the results presented in the main text. In column (4), we allow the effects of precipitation, temperature and wind speed to vary non-linearly, replacing the linear controls with non-parametric indicators for differing ranges (bins) of recorded daily means. ("Yes" denotes that estimates are too numerous to report.) In column (5), we allow a nonlinear effect of the gasoline share. We replace the gasoline share variable with indicators for observations in which the gasoline share: is greater than 0.27 but less than 0.4 (22% of the sample); is greater than 0.40 but less than 0.5 (36% of sample); or is greater than 0.5 (19% of the sample, with a maximum gasoline share of 0.76). The omitted indicator is for observations with a gasoline share lower than 0.27 (23% of sample, with a minimum share of 0.14). In the presence of many controls, we obtain that PM2.5 levels tended to be slightly lower when the proportion of gasoline in the flex-fuel vehicle fleet was higher, though the variation is statistically insignificant (again, all point estimates on the increasing gasoline share indicators are within 1.96 standard errors of zero).

Columns (6) to (8) report robustness checks in which we, respectively: (6) add meteorological conditions observed a day earlier; (7) flexibly allow the effects of cycles (week of the year and day of the week), meteorology, and thermal inversions to vary by measurement site, thus interacting these variables with site fixed effects; and (8) restrict

the sample to non-holiday weekdays, when the mean mass concentration is a higher 14.6 μg m^{-3}. In particular, in column (7), the small increase in R^2, despite more than doubling the number of regressors relative to column (3), suggests that seasonal and meteorological effects on PM2.5 levels are similar across sites.

Finally, columns (9) and (10) test robustness around column (3) specification's site-specific trends, included to capture secular (and site-specific) changes in emissions. Column (9) replaces site-specific trends by year-site fixed effects. Intuitively, this specification examines the co-variation in PM2.5 mass concentration and the gasoline share within a calendar year (e.g., 2011) and site. The estimated coefficient on the gasoline share remains statistically insignificant, now at -1.7 μg m^{-3} with a s.e. of 3.3 μg m^{-3}. Thus estimation precision is lower compared to column (3). Column (10) replaces the year by site fixed effects in column (9) by only site fixed effects, i.e., the intercept for the Pinheiros site is now invariant to year.

To further assess the sensitivity of PM2.5 results, Supplementary Table 2 fixes the vector of explanatory variables for the second-step particle regression model – a somewhat more parsimonious specification with 63 regressors compared to, e.g., 76 regressors in Supplementary Table 1, column (3). Now, however, we consider two variants to the two-step (fuel shares and ambient particles) empirical model. Instrumental-variables estimates, with p_e/p_g instrumenting for $\hat{s}_t^{\text{gas}}$, are reported in columns (2) and (5), with the dependent variable, PM2.5 mass concentration, in μg m^{-3} and its natural logarithm, respectively. Estimates using $s_t^{\text{gas,aggr}}$ to proxy for the fuel mix are reported in columns (3) and (6) (again, PM2.5 in μg m^{-3} and log units, respectively). For comparison, second-step estimates using $\hat{s}_t^{\text{gas}}$ and the same vector of regressors are shown in columns (1) and (4) (μg m^{-3} and log units).

The main message of Supplementary Table 2 is the robustness of the result reported in Table 2, column (2) in the main text, in particular, that variation in the gasoline-ethanol mix has a "somewhat precisely estimated zero" effect on PM2.5 levels. In column (2), raising the penetration of blended gasoline in the flex-fuel light-vehicle fleet from 30% to 80% – the magnitude of variation that occurred within the sample period – is associated with a small and statistically insignificant 0.4 μg m^{-3} reduction in 24-hour PM2.5 levels, with a s.e. of 1.4 μg m^{-3}. In column (3), in-sample variation in the share of blended gasoline

in the entire fleet of light vehicles and motorcycles, from 50% to 80%, is associated with a noisier and statistically insignificant 1.5 μg m^{-3} PM2.5increase, with a s.e. of 1.8 μg m^{-3}.

To provide added perspective on our control variables, we find that mean PM2.5 levels on days in which no precipitation is recorded are higher by 2.6 μg m^{-3} (s.e. 1.0 μg m^{-3}) compared to days with moderately high precipitation rates, averaging between 0.3 and 1 mm $hour^{-1}$ (column (2) estimates, not shown for brevity). Similarly, we find that on days in which a thermal inversion layer with base between 200 and 500 m from the ground level is recorded at 09:00, 24-hour PM2.5 concentrations are higher by 2.0 μg m^{-3} (s.e. 0.8 μg m^{-3}) compared to days in which no thermal inversion is observed at this height.

Finally, the estimates shown in Supplementary Table 2 are robust to restricting the sample to non-holiday weekdays, when road traffic levels and PM2.5 mass concentrations are higher – for brevity, estimates are not reported. Compared to column (1), for example, the coefficient on the gasoline share remains insignificant and is estimated with less precision in the weekday-only sample, changing from -0.1 μg m^{-3} (s.e. 1.8 μg m^{-3}) to -0.9 μg m^{-3} (s.e. 2.7 μg m^{-3}). Similarly, Supplementary Table 2 estimates are robust to adding site-specific indicators for wind direction, as in the less parsimonious specification shown in Supplementary Table 1, column (3). Specifically, we include a set of four dummy variables that turn on when wind is blowing to a site from each of four quadrants (clockwise 0 to 90 degrees, 90 to 180 degrees, etc) and at a minimum wind speed of 0.5 m s^{-1}; thus, the omitted category indicates still air. In column (1), the coefficient on the gasoline share changes slightly to 0.2 μg m^{-3} (s.e. 2.0 μg m^{-3}) – again estimates are not reported. Estimates are also robust to additionally controlling for ridership on diesel buses in the metropolis' public transport system (PM2.5 in Figure 3 to be compared to Figure 1 in the main text), as well as the real price of diesel.

Supplementary Table 1. Changes to filter-based 24-hour PM2.5 mass concentrations from variation in the gasoline-ethanol fuel mix.

Dependent variable: PM2.5 (μg m^{-3})	(1)	(2)	(3)	(4)	(5)	(6)	(7)	(8)	(9)	(10)
Specification: Adding more/alternative controls:	No controls	Fixed effects	Meteor. & traffic	Nonlinear meteor.	Nonlinear share	1-day lag meteor.	Site-spec. meteor.	Weekday only	Site-spec. year FEs	No trend
Share of Gasoline E20/E25 over Ethanol E100: Flex fleet 30 to 80%	-1.2	2.3	0.1	1.0		0.2	0.0	-1.2	-1.7	-1.3
	(1.8)	(2.7)	(2.0)	(2.5)		(2.2)	(2.1)	(3.6)	(3.3)	(1.9)
Indicator for 0.27 ≤ Gasoline share < 0.40					-1.2					
					(1.7)					
Indicator for 0.40 ≤ Gasoline share < 0.50					-1.8					
					(2.1)					
Indicator for 0.50 < Gasoline share					-0.7					
					(1.7)					
**Control variables**										
Site-specific linear trend or year fixed effect		Site&trend	Site&trend	Site&trend	Site&trend	Site&trend	Site&trend	Site&trend	Site&year	Only site
Week-of-year fixed effects		Yes	Yes	Yes	Yes	Yes	Yes	Yes	Yes	Yes
Day-of-week fixed effects		Yes	Yes	Yes	Yes	Yes	Yes	Yes	Yes	Yes
Radiation (per 100 W m^{-2})			-0.2	-0.2	-0.1	-0.3	Yes	1.1	-0.3	-0.3
			(0.7)	(0.7)	(0.7)	(0.7)		(1.0)	(0.7)	(0.7)
Temperature (per 1 °C or several indicators)			1.3	Yes	1.3	1.5	Yes	1.1	1.3	1.3
			(0.2)		(0.2)	(0.4)		(0.2)	(0.2)	(0.2)
Humidity (per 10 pct)			-0.8	-1.3	-0.8	-0.3	Yes	-0.7	-0.7	-0.9
			(0.7)	(0.7)	(0.6)	(0.8)		(0.9)	(0.7)	(0.6)
Wind speed (per 1 m s^{-1} or several indicators)			-5.8	Yes	-6.0	-5.1	Yes	-6.6	-6.4	-5.8
			(1.1)		(1.1)	(1.3)		(1.7)	(1.1)	(1.1)
Precipitation (per 1 mm h^{-1} or several indicators)			-0.4	Yes	-0.4	-0.3	Yes	0.4	-0.5	-0.4
			(0.6)		(0.6)	(0.6)		(0.8)	(0.6)	(0.6)
Thermal inversion indicators (base at 0-199 m, 200-499 m)			Yes	Yes	Yes	Yes	Yes	Yes	Yes	Yes
Wind direction quadrant & Wind speed>0.5 m s^{-1} indicators (several)			Yes	Yes	Yes	Yes	Yes	Yes	Yes	Yes
Road traffic congestion indicators (several)			Yes	Yes	Yes	Yes	Yes	Yes	Yes	Yes
Site-specific intercept after southern beltway opening (several)			Yes	Yes	Yes	Yes	Yes	Yes	Yes	Yes
R^2	0.3%	29.0%	74.2%	73.1%	74.5%	74.5%	76.1%	79.7%	76.0%	73.4%
Number of observations	511	511	511	511	511	511	511	324	511	511
Number of regressors	2	48	76	82	78	81	167	68	88	73
Mean value of dependent variable	13.8	13.8	13.8	13.8	13.8	13.8	13.8	14.6	13.8	13.8

Notes: Coefficients and standard errors (in parentheses). An observation is a date-site pair. The sample includes 3 monitoring sites (Cerqueira César, Ibirapuera, Pinheiros) and all days of the week. The sample period is November 1, 2008 to May 31, 2013, excluding the colder months of June to September. The dependent variable is the mean PM2.5 mass concentration on a given date and site. Ordinary Least Squares estimates. Standard errors are calculated by bootstrapping (200 samples each): (i) the consumer-level fuel choice data, to account for sampling variation in the predicted gasoline share, and (ii) the pollutant-meterology-traffic data, clustering by date.

Supplementary Table 2. Changes to filter-based 24-hour PM2.5 mass concentrations from variation in the gasoline-ethanol fuel mix.

Dependent variable:	PM2.5 (μg m^{-3})			Log PM2.5 (log points)		
	(1)	(2)	(3)	(4)	(5)	(6)
Specification: 2nd step model based on:	Share in the flex fleet	Share in the flex fleet	Share in the aggreg. fleet	Share in the flex fleet	Share in the flex fleet	Share in the aggreg. fleet
Estimation:	OLS + bootstr.	2SLS	OLS	OLS + bootstr.	2SLS	OLS
Share of Gasoline E20/E25 over Ethanol E100	Flex 30 to 80%:	Flex 30 to 80%:	Aggr. 50 to 80%:	Flex 30 to 80%:	Flex 30 to 80%:	Aggr. 50 to 80%:
	-0.1	-0.4	1.5	-0.01	-0.04	0.15
	(1.8)	(1.4)	(1.8)	(0.16)	(0.11)	(0.17)
**Control variables**						
Site-specific linear trend	Yes	Yes	Yes	Yes	Yes	Yes
Week-of-year fixed effects	Yes	Yes	Yes	Yes	Yes	Yes
Day-of-week fixed effects	Yes	Yes	Yes	Yes	Yes	Yes
Radiation (per 100 W m^{-2} or log)	0.2	0.2	0.0	0.08	0.09	0.06
	(1.0)	(0.7)	(0.7)	(0.14)	(0.09)	(0.10)
Temperature (per 1 ^{o}C or log)	1.0	1.0	1.0	2.02	2.03	1.99
	(0.2)	(0.2)	(0.2)	(0.47)	(0.35)	(0.37)
Humidity (per 10 pct or log)	-0.6	-0.6	-0.7	-0.46	-0.44	-0.51
	(0.8)	(0.6)	(0.6)	(0.46)	(0.35)	(0.38)
Wind speed (per 1 m s^{-1} or log)	-6.0	-6.0	-6.1	-0.68	-0.68	-0.69
	(1.2)	(0.8)	(0.9)	(0.15)	(0.10)	(0.10)
Precipitation indicators (several)	Yes	Yes	Yes	Yes	Yes	Yes
Thermal inversion indicators (base at 0-199 m, 200-499 m)	Yes	Yes	Yes	Yes	Yes	Yes
Road traffic congestion indicators (several)	Yes	Yes	Yes	Yes	Yes	Yes
Site-specific intercept after southern beltway opening (several)	Yes	Yes	Yes	Yes	Yes	Yes
R^2	70.9%	70.9%	71.0%	71.6%	71.6%	71.8%
Number of observations	511	511	511	511	511	511
Number of regressors	63	63	63	63	63	63
Mean value of dependent variable	13.8	13.8	13.8	2.5	2.5	2.5

Notes: Coefficients and standard errors (in parentheses). An observation is a date-site pair. The sample includes 3 monitoring sites (Cerqueira César, Ibirapuera, Pinheiros) and all days of the week. The sample period is November 1, 2008 to May 31, 2013, excluding the colder months of June to September. The dependent variable is the mean PM2.5 mass concentration on a given date and site (columns 1 to 3), or its logarithm (columns 4 to 6). Radiation, temperature, humidity, and wind speed in the recorded unit (columns 1 to 3), or its logarithm (columns 4 to 6). Two-Stage Least Squares estimates in columns 2 and 5, with the median ethanol-to-gasoline price ratio across pumping stations instrumenting for the predicted gasoline share in the flex-fuel fleet. Ordinary Least Square estimates in the remaining columns. In columns 1 and 4, standard errors are calculated by bootstrapping (200 samples each): (i) the consumer-level fuel choice data, to account for sampling variation in the predicted gasoline share, and (ii) the pollutant-meterology-traffic data, clustering by date. In columns 3 and 6, the gasoline share is calculated from reported aggregate wholesaler shipments that serve the entire light-vehicle fleet (including both flex-fuel and single-fuel engines, and motorcycles).

Supplementary Note 2. PM2.5 concentrations based on beta-continuous measurements. Supplementary Table 3 provides further sensitivity analysis with regard to the estimates reported in Table 2, column (2) in the main text, but now examining the hourly series for PM2.5 mass concentrations. The environmental authority began measuring PM2.5 with a beta continuous analyzer only in January 2011, and the first site was Congonhas, right by a busy road and in close proximity to Congonhas airport. Hourly sampling at two other sites, Pinheiros and IPEN-USP, started in late 2011 and 2012, respectively. We take advantage of the high-frequency PM2.5 measurements and restrict the sample to the morning hours of 07:00 and 11:00 inclusive, in which fresh emissions from vehicular traffic (and secondary aerosol formed from these primary emissions) are likely to be most important. An observation is a site-date pair, but now the dependent variable is the mean PM2.5 concentration (or its logarithm) measured between 07:00 and 11:00, rather than a 24-hour mean.

The mean concentration in the sample of 1310 site-date observations (every day, 2011/2012 to 2013, excluding the colder months of June to September) is 18.8 μg m^{-3}, 37% higher than the Cerqueira César, Pinheiros, Ibirapuera filter measurement average (Supplementary Table 1), underscoring Congonhas' localized emissions sources (roads and inner-city airport). Again, we find no statistically significant relationship between the gasoline share of the fuel mix and morning PM2.5 concentrations, whether we consider the baseline second-step particle regression model (Supplementary Table 3, columns (1) and (4), using $\hat{s}_t^{gas}$ and a bootstrap procedure to account for sampling variation in the first step) or its variants (shown in the remaining columns). Estimated coefficients on the gasoline share are larger in magnitude, and more negative, than those reported in the main text (Table 2, column (2)), but they are also noisier estimates. For example, in column (1), the estimated coefficient on the gasoline share in the flex-fuel vehicle fleet, for a 30% to 80% rise in share, is -2.6 μg m^{-3} with a s.e. of 3.3 μg m^{-3} in Supplementary Table 3, compared to -0.1 μg m^{-3} with a s.e. of 1.8 μg m^{-3} in Supplementary Table 2. Lower precision is likely due in part to the shorter sample period, starting in 2011 rather than 2008.

We also obtain lower R^2, of about 50%, compared to 70% earlier. To the extent that unobserved time-varying determinants of PM2.5 were present locally at Congonhas, and happened to correlate with the 2011 high-priced-ethanol episode, this would violate the

identifying assumptions (Methods). With a view to reducing this possibility further, we control for the number of departing flights from Congonhas airport (and we have experimented with other airport traffic conditions). We also note (though do not report) that, on restricting the sample to non-holiday weekdays, the coefficient on the gasoline share changes only slightly and is estimated less precisely. For example, the weekday-only sample yields a gasoline share coefficient of -3.3 μg m^{-3} (s.e. 4.2 μg m^{-3}) compared to Supplementary Table 3, column (1)'s -2.6 μg m^{-3} (s.e. 3.3 μg m^{-3}) when all days of the week are included.

We again find that morning PM2.5 concentrations rise when higher temperatures and thermal inversions are recorded (contemporaneously), and fall in windy and rainy weather. For example, in column (1), a +1 °C increase in (morning mean) temperature is associated with a 0.8 μg m^{-3} increase in PM2.5, with a s.e. of 0.2 μg m^{-3}; a +1 m s^{-1} increase in wind speed is associated with a 8.7 μg m^{-3} decrease in PM2.5, with a s.e. of 0.9 μg m^{-3}. We also find that morning PM2.5 levels are negatively associated with relative humidity.

Using this high-frequency data source and restricting the sample to non-holiday weekdays, Supplementary Fig. 9 plots 95% CI for the gasoline share estimated in hour-by-hour regressions of PM2.5 concentrations, that is, separate regressions by hour of the day, 0:00 to 23:00. We follow the model in Supplementary Table 3, column (2). We are unable to reject the hypothesis of a zero effect of gasoline versus ethanol use on PM2.5 concentrations at any point in the diurnal cycle.

Supplementary Figure 9. Estimated changes in PM2.5 concentrations over the diurnal cycle associated with a rise in blended gasoline (E20/E25) use in the flex-fuel light-vehicle fleet from 30 to 80 percentage points.

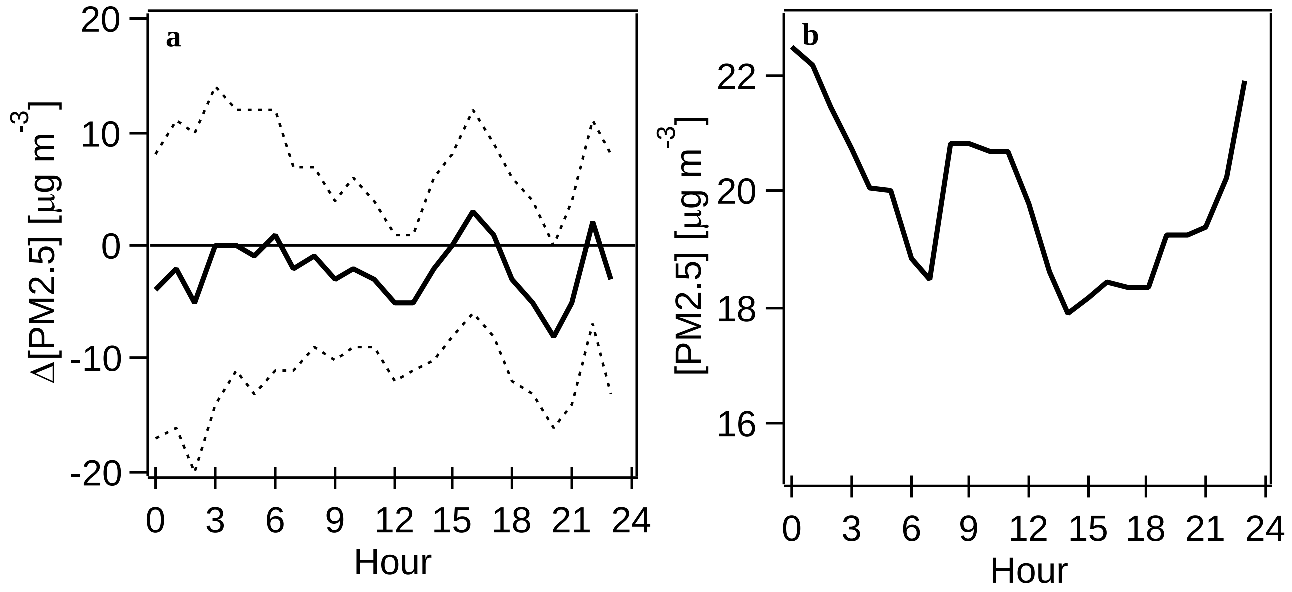

(**a**) 95% CI of the estimated change, in μg m^{-3}, and (**b**) mean concentration, by hour, in the sample of non-holiday weekdays, based on beta-continuous monitoring at Congonhas, Pinheiros and IPEN-USP sites.

Supplementary Table 3. Changes to beta-based morning PM2.5 mass concentrations from variation in the gasoline-ethanol fuel mix.

Dependent variable:	PM2.5 (µg m^{-3})			Log PM2.5 (log points)		
	(1)	(2)	(3)	(4)	(5)	(6)
Specification: 2nd step model based on:	Share in the flex fleet	Share in the flex fleet	Share in the aggreg. fleet	Share in the flex fleet	Share in the flex fleet	Share in the aggreg. fleet
Estimation:	OLS + bootstr.	2SLS	OLS	OLS + bootstr.	2SLS	OLS
Share of Gasoline E20/E25 over Ethanol E100	Flex 30 to 80%:	Flex 30 to 80%:	Aggr. 50 to 80%:	Flex 30 to 80%:	Flex 30 to 80%:	Aggr. 50 to 80%:
	-2.6	-1.6	-2.9	-0.13	-0.09	-0.17
	(3.3)	(3.0)	(2.5)	(0.16)	(0.15)	(0.13)
**Control variables**						
Site-specific linear trend	Yes	Yes	Yes	Yes	Yes	Yes
Week-of-year fixed effects	Yes	Yes	Yes	Yes	Yes	Yes
Day-of-week fixed effects	Yes	Yes	Yes	Yes	Yes	Yes
Radiation (per 100 W m^{-2} or log)	0.0	0.0	0.1	0.10	0.10	0.10
	(0.4)	(0.4)	(0.4)	(0.04)	(0.04)	(0.04)
Temperature (per 1 ^{o}C or log)	0.8	0.8	0.7	1.02	1.03	1.01
	(0.2)	(0.2)	(0.2)	(0.22)	(0.20)	(0.20)
Humidity (per 10 pct or log)	-1.6	-1.6	-1.6	-0.30	-0.30	-0.30
	(0.6)	(0.5)	(0.5)	(0.18)	(0.16)	(0.17)
Wind speed (per 1 m s^{-1} or log)	-8.7	-8.7	-8.8	-0.55	-0.55	-0.55
	(0.9)	(0.8)	(0.8)	(0.06)	(0.06)	(0.06)
Precipitation indicators (several)	Yes	Yes	Yes	Yes	Yes	Yes
Thermal inversion indicators (base at 0-199 m, 200-499 m)	Yes	Yes	Yes	Yes	Yes	Yes
Road traffic congestion indicators (several)	Yes	Yes	Yes	Yes	Yes	Yes
Number of aircraft departing, effect by day type (several)	Yes	Yes	Yes	Yes	Yes	Yes
R^2	48.4%	48.4%	46.0%	48.5%	48.5%	48.5%
Number of observations	1310	1310	1310	1306	1306	1306
Number of regressors	67	67	67	67	67	67
Mean value of dependent variable	18.8	18.8	18.8	2.8	2.8	2.8

Notes: Coefficients and standard errors (in parentheses). An observation is a date-site pair. The sample includes 3 monitoring sites (Congonhas, Pinheiros, IPEN-USP) and all days of the week. The sample period is January 1, 2011 to May 31, 2013, excluding the colder months of June to September. The dependent variable is the mean mass concentration between 07:00 and 11:00 on a given date and site (columns 1 to 3), or its logarithm (columns 4 to 6). Radiation, temperature, humidity, and wind speed in the recorded unit (columns 1 to 3), or its logarithm (columns 4 to 6). Two-Stage Least Squares estimates in columns 2 and 5, with the median ethanol-to-gasoline price ratio across pumping stations instrumenting for the predicted gasoline share in the flex-fuel fleet. Ordinary Least Square estimates in the remaining columns. In columns 1 and 4, standard errors are calculated by bootstrapping (200 samples each): (i) the consumer-level fuel choice data, to account for sampling variation in the predicted gasoline share, and (ii) the pollutant-meterology-traffic data, clustering by date. In columns 3 and 6, the gasoline share is calculated from reported aggregate wholesaler shipments that serve the entire light-vehicle fleet (including both flex-fuel and single-fuel engines, and motorcycles).

Supplementary Note 3. Ozone concentrations. Supplementary Table 4 provides sensitivity analysis with regard to the estimates reported in Table 2, column (5) in the main text. An observation is a site-date pair, and the dependent variable is the mean early-afternoon ozone concentration (or its logarithm) measured between 12:00 and 16:00. The mean concentration in the sample of 13,203 site-date observations (every day, 2008 to 2013, excluding the colder months of June to September) is 72.2 μg m^{-3}.

Supplementary Fig. 10 shows results for ozone alongside those for PM2.5 (Supplementary Notes 1 and 2). In contrast to PM2.5, the effect of the light-vehicle fuel mix on ozone levels is significant, with ozone levels falling with gasoline use[3].

Supplementary Figure 10. Estimated changes in PM2.5 and O_3 mass concentrations from raising blended gasoline (E20/E25) use in the flex-fuel light-vehicle fleet from 30 to 80 percentage points.

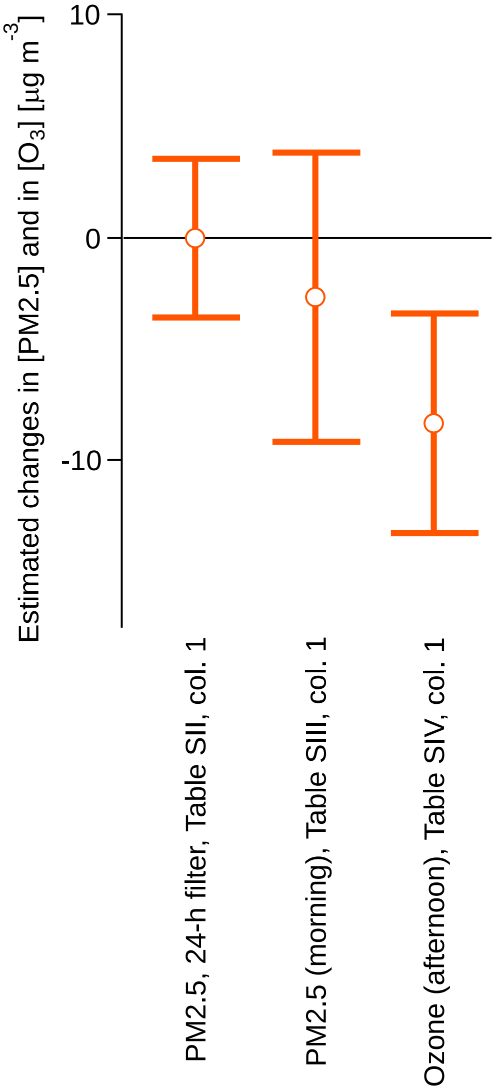

95% confidence intervals (CI) calculated from: estimates for 24-hour PM2.5, in µg m^{-3}, shown in Supplementary Table 2, column (1), based on 24-hour filters collected at Cerqueira César, Ibirapuera and Pinheiros sites between 2008 and 2013; estimates for morning PM2.5, in µg m^{-3}, shown in Supplementary Table 3, column (1), based on beta-continuous monitoring at Congonhas, Pinheiros and IPEN-USP sites between 2011/2012 and 2013; and estimates for afternoon ozone, in µg m^{-3}, shown in Supplementary Table 4, column (1), based on ultraviolet-continuous monitoring at 12 sites across the São Paulo metropolitan area between 2008 and 2013.

Supplementary Table 4. Changes to early-afternoon ozone mass concentrations from variation in the gasoline-ethanol fuel mix.

Dependent variable:	Ozone (μg m^{-3})			Log Ozone (log points)		
	(1)	(2)	(3)	(4)	(5)	(6)
Specification (2nd step model):	Share in the flex fleet	Share in the flex fleet	Share in the aggreg. fleet	Share in the flex fleet	Share in the flex fleet	Share in the aggreg. fleet
Estimation:	OLS + bootstr.	2SLS	OLS	OLS + bootstr.	2SLS	OLS
Share of Gasoline E20/E25 over Ethanol E100	Flex 30 to 80%:	Flex 30 to 80%:	Aggr. 50 to 80%:	Flex 30 to 80%:	Flex 30 to 80%:	Aggr. 50 to 80%:
	-8.3	-9.4	-4.3	-0.16	-0.17	-0.14
	(2.5)	(2.2)	(2.8)	(0.04)	(0.04)	(0.05)
**Control variables**						
Site-specific linear trend	Yes	Yes	Yes	Yes	Yes	Yes
Week-of-year fixed effects	Yes	Yes	Yes	Yes	Yes	Yes
Day-of-week fixed effects	Yes	Yes	Yes	Yes	Yes	Yes
Radiation (per 100 W m^{-2} or log)	4.2	4.2	4.0	0.35	0.35	0.34
	(0.4)	(0.4)	(0.4)	(0.03)	(0.03)	(0.04)
Temperature (per 1 ^{o}C or log)	3.1	3.1	3.1	1.49	1.49	1.48
	(0.2)	(0.2)	(0.2)	(0.09)	(0.09)	(0.11)
Humidity (per 10 pct or log)	-4.9	-4.9	-5.1	-0.35	-0.35	-0.37
	(0.6)	(0.6)	(0.6)	(0.06)	(0.05)	(0.06)
Wind speed (per 1 m s^{-1} or log)	-13.2	-13.2	-12.9	-0.21	-0.21	-0.20
	(1.1)	(1.1)	(1.1)	(0.04)	(0.03)	(0.04)
Precipitation indicators (several)	Yes	Yes	Yes	Yes	Yes	Yes
Thermal inversion indicators (base at 0-199 m, 200-499 m)	Yes	Yes	Yes	Yes	Yes	Yes
Morning road traffic congestion indicators (several)	Yes	Yes	Yes	Yes	Yes	Yes
Site-specific intercept after southern beltway opening (several)	Yes	Yes	Yes	Yes	Yes	Yes
R^2	70.7%	70.6%	70.5%	71.5%	71.5%	71.4%
Number of observations	13203	13203	13203	13203	13203	13203
Number of regressors	96	96	96	96	96	96
Mean value of dependent variable	72.2	72.2	72.2	4.1	4.1	4.1

Notes: Coefficients and standard errors (in parentheses). An observation is a date-site pair. The sample includes 12 monitoring sites and all days of the week. The sample period is November 1, 2008 to May 31, 2013, excluding the colder months of June to September. The dependent variable is the mean concentration between 12:00 and 16:00 on a given date and site (columns 1 to 3), or its logarithm (columns 4 to 6). Radiation, temperature, humidity, and wind speed in the recorded unit (columns 1 to 3), or its logarithm (columns 4 to 6). Thermal inversion and traffic as recorded between 07:00 and 11:00. Two-Stage Least Squares estimates in columns 2 and 5, with the median ethanol-to-gasoline price ratio across pumping stations instrumenting for the predicted gasoline share in the flex-fuel fleet. Ordinary Least Square estimates in the remaining columns. In columns 1 and 4, standard errors are calculated by bootstrapping (200 samples each): (i) the consumer-level fuel choice data, to account for sampling variation in the predicted gasoline share, and (ii) the pollutant-meterology-traffic data, clustering by date. In columns 3 and 6, the gasoline share is calculated from reported aggregate wholesaler shipments that serve the entire light-vehicle fleet (including both flex-fuel and single-fuel engines, and motorcycles).

Supplementary Note 4. Submicron particles and BC. Supplementary Table 5 to 8 provide sensitivity analysis with regard to the submicron particle and BC results presented in Tables 2 and 3 in the main text. Some notes on these regression models are in order. Since the sampling period is short of one year, we cannot employ week-of-year fixed effects. Importantly, a full set of 52(-1) week-of-year intercepts would not only absorb any seasonal variation in the modeled submicron parameter that might remain unexplained beyond the other included controls (such as meteorology), but they would also subsume the temporal source of fuel mix variation that is our main variable of interest. Thus, instead of week, we include coarser quarter-of-year fixed effects (January-March, April-June, etc.), and exploit within-quarter fuel mix variation.

In addition to using the full sample that comprises three seasons (spring, summer, fall) and employing quarter-of-year fixed effects, we also consider a shorter subsample. In this alternative specification, we restrict the sample to the set of summer and fall months from late January to May 2011, during which seasonal variation is less pronounced and arguably "monotonic." We also include a linear trend. Of note, this shorter sample still encompasses the second and most pronounced episode of ethanol price variation, marked by a rise followed by a drop in relative prices. The shorter sample also excludes the early January vacation period in which vehicle emissions may differ substantially. We refer to this shorter sample as the more seasonally homogeneous sample.

In sum, our regression analysis uses both the full sample of measurements and, separately, the shorter mid-summer to fall sample in which seasonal variation is less pronounced. An ideal dataset would cover a multiyear period and allow us to include finer week-of-year seasonal controls, as is the case for officially monitored PM2.5, but such data are not available in the submicron mode. We thus caution that, in what follows, if judged by the empirical model only, one can interpret our estimated coefficients on the gasoline share as statistical associations rather than causal effects. During a sampling period short of an annual cycle, one might worry that observed changes in the fuel mix co-vary with omitted seasonal (or other time-varying) determinants of particle pollution that remain in the residual ε_{lt}; this would violate the identifying assumption $E[\text{fuel\_mix}_t \varepsilon_{lt} | \tilde{X}_{lt}] = 0$, where the tilde denotes the absence of fine seasonal controls in the vector of controls, X.

Three aspects give us confidence that our empirical findings are not mere statistical artefacts. First, we obtain differential results for different particle size ranges and times of day, namely the nucleation versus the Aitken and accumulation modes, in Supplementary Table 5; and the 7-100 nm versus 100-800 nm size ranges during the peak hours of morning travel but not outside these hours, as presented in Supplementary Tables 6 to 8 and Supplementary Figs. 13 and 14. Second, when restricting our sample to dates between late January and May in which meteorology may vary seasonally but "monotonically," as mid-summer conditions evolve into those that characterize mid-fall, the ultrafine particle levels we uncover after correcting for confounding variation, including trending variation, *fluctuate* in lockstep with the penetration of gasoline: first up, until the beginning of April, followed by down (Figure 2d in the main text). The third factor that strengthens our findings is that they are consistent with controlled emissions studies and laboratory experiments cited in the main text.

Supplementary Table 5 provides sensitivity analysis with regard to the estimates reported in Table 3 in the main text. We further examine variation in the CPC particle number concentration, in columns (1) and (2); in the contribution of nucleation, Aitken and accumulation modes, in columns (3) to (8); and in the BC mass concentration, in columns (9) and (10). An observation is a date in the full period that the field campaign lasted, and variables are 24-hour means for the indicated parameters. The odd-numbered columns report second-step model estimates for which we account for sampling error in the gasoline share ($\hat{s}_t^{\text{gas}}$) via a bootstrap procedure. In the even-numbered columns, we alternatively account for sampling error in the gasoline share using 2SLS, with the ethanol-gasoline price ratio as an instrument for $\hat{s}_t^{\text{gas}}$. We include quarter-of-year fixed effects, and note that our estimates are robust to adding, on top of the quarter dummies, a linear trend. Estimates are also robust to additionally controlling for ridership on diesel buses in the metropolis' public transport system (BC in Figure 3 to be compared to Figure 1 in the main text), as well as the real price of diesel.

Columns (1) and (2) show a statistically insignificant association between the gasoline share and the particle number concentration as measured by the CPC. To see this in column (1), for example, the point estimate on the gasoline share, 1335 cm^{-3} (for a 30% to 80% shift in the share of the flex fleet) is within 1.96 standard errors (1.96×965=1892

cm^{-3}) of 0 cm^{-3}. Over the dates in the sample, the particle count is decreasing in ambient temperature and humidity. Similar to effects on PM2.5 mass concentration, stronger winds also have a significant downward effect on particle number concentrations.

Columns (3) to (8) report a statistically significant and positive association between the gasoline share and the contribution of nucleation mode particles to the aerosol particle size distribution, but an insignificant association in the Aitken and accumulation modes, consistent with Table 3 in the main text. Estimates indicate differential meteorological dependence across the different modes. Temperature has a negative and statistically significant effect on the nucleation and Aitken modes, whereas an insignificantly positive effect on the accumulation mode. Nucleation mode particles are most sensitive to humidity: a 10-percentage point increase in relative humidity is associated with a 2048 cm^{-3} drop in peak nucleation dN/dlogDp, equivalent to 23% of the mean value of the parameter across dates in the sample. Wind speed affects all modes, with a particularly large proportionate effect on the accumulation mode.

Columns (9) and (10), similar to Table 3 in the main text, report a statistically insignificant association between the gasoline share and BC concentration. BC concentrations are negatively associated with humidity and wind speed, and positively associated with the observation of thermal inversion.

Supplementary Fig. 11 reports 95% CI for the gasoline/particle count association, taking as dependent variable the hour-specific particle count measure, in hour-by-hour regressions from 0:00 to 23:00. We restrict the sample to non-holiday weekdays. Similarly, Supplementary Fig. 12 reports 95% CI for the gasoline/BC concentration association, taking as dependent variable the hour-specific BC concentration measure. In both cases, the same result obtained for the 24-hour means – the absence of a statistically significant relationship with the fuel mix – holds over the diurnal cycle. The figures illustrate how each dependent variable – particle number concentration or BC concentration in ambient air – evolves during a typical workday. In particular, from 05:00 to 09:00, both the particle count and the BC concentration double, falling thereafter through the late morning and afternoon hours. BC concentrations reverse course and start rising around 18:00, consistent with the evening commute. The diurnal correlation with observed traffic congestion is noteworthy and, as a partial marker of road traffic, can help interpret the positive and

significant association between gasoline and 7-100 nm particle levels in the morning hours (Figure 4 in the main text).

For perspective, the highly temporally and spatially resolved data indicate that, averaged across all non-holiday weekdays in the BC sample, road traffic congestion within a 2 km radius of the IPEN-USP site nearby to where BC was monitored, increased monotonically from 05:00, peaking at 09:00 with a total extension of 0.82 km of congested road segments. Similarly, across the city's monitored road grid, traffic congestion aggregated up to the west region of the city, or aggregated up further to the entire city, both peak at 09:00, at 27 km and 92 km of extension, respectively.

Supplementary Figure 11. Estimated changes in the aerosol particle number concentration over the diurnal cycle associated with a rise in blended gasoline (E20/E25) use in the flex-fuel light-vehicle fleet from 30 to 80 percentage points.

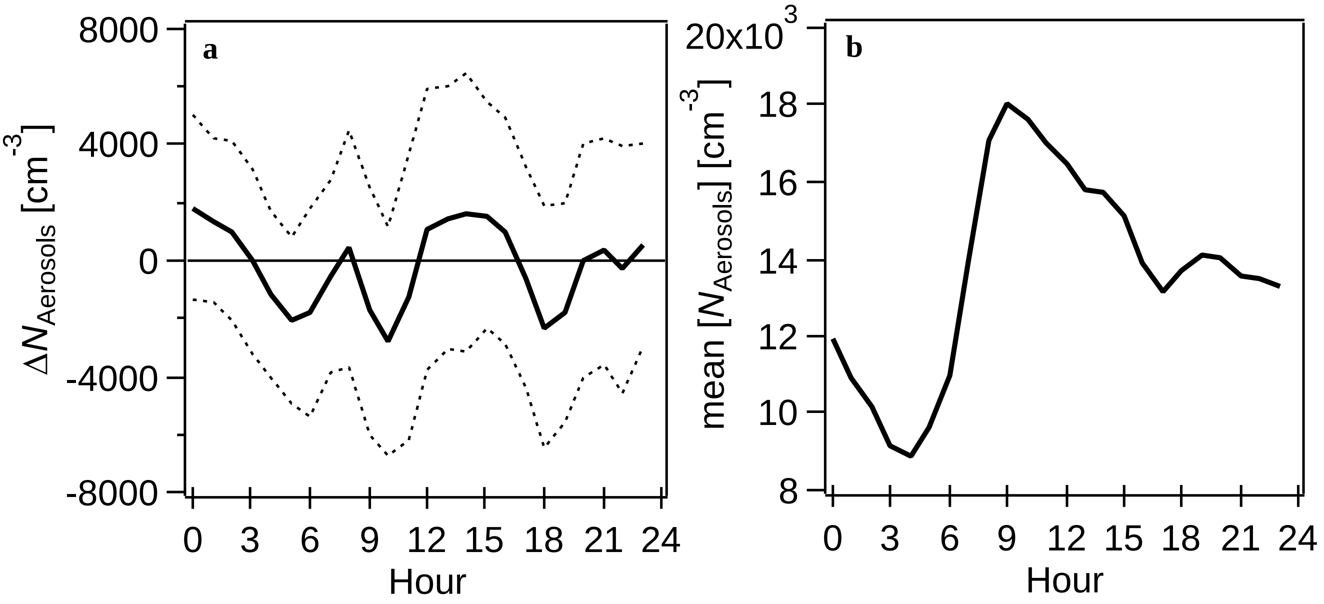

(**a**) 95% CI of the estimated change, in cm^{-3}, and (**b**) mean concentration, by hour, in the sample of non-holiday weekdays as measured by the CPC.

Supplementary Figure 12. Estimated changes in the BC concentration over the diurnal cycle associated with a rise in blended gasoline (E20/E25) use in the flex-fuel light-vehicle fleet from 30 to 80 percentage points.

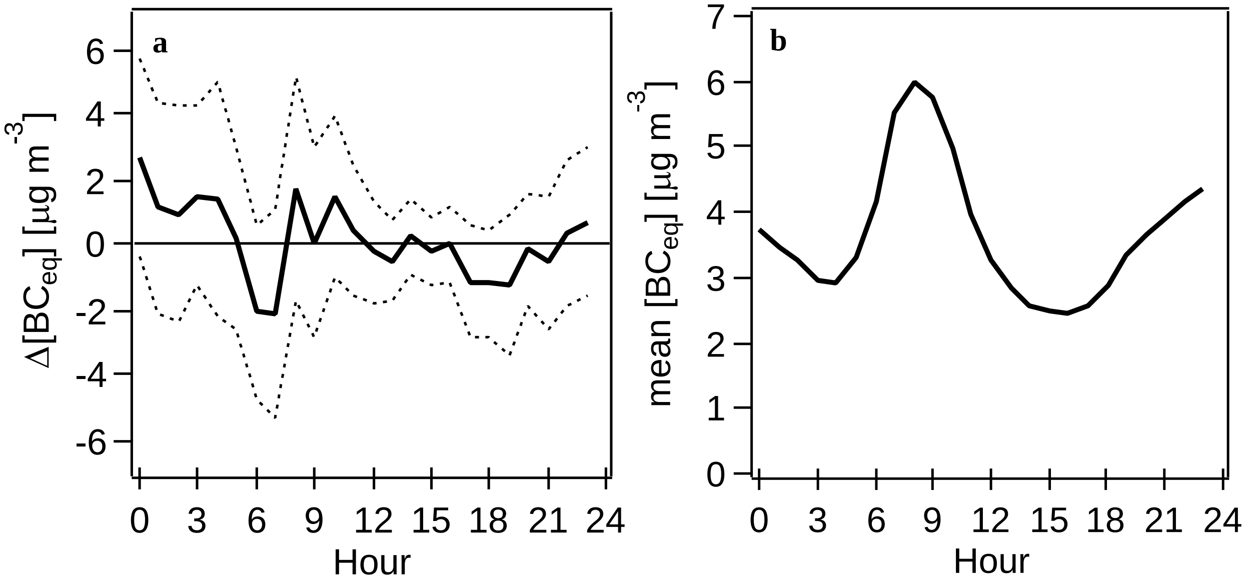

(**a**) 95% CI of the estimated change, in μg m^{-3}, and (**b**) mean concentration, by hour, in the sample of non-holiday weekdays.

Supplementary Table 5. Associations between 24-hour mean submicron particle characteristics and the gasoline-ethanol fuel mix.

Dependent variable:	Particle count (cm^{-3})		Nucleation (peak, dN/dlogDp)		Aitken (dN/dlogDp)		Accumulation (dN/dlogDp)		BC (μg m^{-3})	
	(1)	(2)	(3)	(4)	(5)	(6)	(7)	(8)	(9)	(10)
Specification (2nd step model):	Share in the flex fleet	Share in the flex fleet	Share in the flex fleet	Share in the flex fleet	Share in the flex fleet	Share in the flex fleet	Share in the flex fleet	Share in the flex fleet	Share in the flex fleet	Share in the flex fleet
Estimation:	OLS + bootstr.	2SLS	OLS + bootstr.	2SLS	OLS + bootstr.	2SLS	OLS + bootstr.	2SLS	OLS + bootstr.	2SLS
Share of Gasoline E20/E25 over Ethanol E100: Flex 30 to 80%	1335	1359	2357	2352	460	490	513	499	0.1	0.1
	(965)	(913)	(796)	(799)	(413)	(420)	(420)	(445)	(0.7)	(0.7)
**Control variables**										
Quarter-of-year fixed effects	Yes	Yes	Yes	Yes	Yes	Yes	Yes	Yes	Yes	Yes
Day-of-week fixed effects	Yes	Yes	Yes	Yes	Yes	Yes	Yes	Yes	Yes	Yes
Radiation (per 100 W m^{-2})	178	178	-568	-568	271	271	98	98	0.1	0.1
	(461)	(404)	(339)	(305)	(181)	(178)	(130)	(114)	(0.2)	(0.1)
Temperature (per 1 ^{o}C)	-509	-511	-382	-382	-123	-125	43	44	0.0	0.0
	(141)	(126)	(118)	(106)	(58)	(51)	(43)	(42)	(0.0)	(0.0)
Humidity (per 10 pct)	-2083	-2086	-2048	-2047	-545	-549	-56	-54	-0.3	-0.3
	(406)	(355)	(341)	(283)	(190)	(164)	(115)	(112)	(0.1)	(0.1)
Wind speed (per 1 m s^{-1})	-4202	-4206	-990	-989	-1775	-1780	-1251	-1249	-2.5	-2.5
	(592)	(550)	(461)	(499)	(281)	(264)	(213)	(193)	(0.2)	(0.2)
Precipitation indicators (several)	Yes	Yes	Yes	Yes	Yes	Yes	Yes	Yes	Yes	Yes
Thermal inversion indicators (base at 0-199 m, 200-499 m)	Yes	Yes	Yes	Yes	Yes	Yes	Yes	Yes	Yes	Yes
Road traffic congestion indicators (several)	Yes	Yes	Yes	Yes	Yes	Yes	Yes	Yes	Yes	Yes
R^2	64.1%	64.1%	50.8%	50.8%	48.4%	48.4%	47.3%	47.3%	69.6%	68.5%
Number of observations	155	155	198	198	198	198	198	198	228	228
Number of regressors	25	25	25	25	25	25	25	25	24	24
Mean value of dependent variable	12753	12753	8755	8755	3320	3320	1494	1494	3.3	3.3

Notes: An observation is a date. Sampling at one site in the University of São Paulo campus. Sample periods are: November 2010 to September 2011 (columns 1 and 2); October 2010 to September 2011 (columns 3 to 8); and October 2010 to April 2011 followed by August 2012 to November 2012 (columns 9 and 10) . The sample excludes the colder months of June to September and includes all days of of the week. The dependent variable is the mean measure, in the unit indicated, between 00:00 and 23:00 on a given date. Radiation, temperature, humidity, and wind speed in the recorded unit. Ordinary Least Squares estimates in the odd-numbered columns, with standard errors calculated by bootstrapping (200 samples each): (i) the consumer-level fuel choice data, to account for sampling variatior in the predicted gasoline share, and (ii) the pollutant-meterology-traffic data, clustering by date. Two-Stage Least Squares estimates in the even-numbered columns, with the median ethanol-to-gasoline price ratio across pumping stations instrumenting for the predicted gasoline share.

Supplementary Note 5. Hour-by-hour 7-100 nm and 100-800 nm size ranges. Supplementary Tables 6 and 7 provide sensitivity analysis with regard to the hour-specific results for the 7-100 nm and 100-800 nm size ranges presented in Table 2 and Figure 4 in the main text. Each table consists of four panels. Panels A, B and D report second-step model estimates with a bootstrap procedure to correct for sampling error in the gasoline share, $\hat{s}_t^{\text{gas}}$. Panel C reports 2SLS estimates, with the ethanol-gasoline price ratio as an instrument for $\hat{s}_t^{\text{gas}}$. Relative to panel A, panel B restricts the sample to non-holiday weekdays, i.e., we drop weekends and public holidays, when economic activity and commuting levels in particular drop, as do PM2.5 concentrations. Relative to panel B, panel D controls for wind direction.

For each hour-specific regression, shown in a different row, we report only the estimated coefficient and standard error on the gasoline share, $\hat{s}_t^{\text{gas}}$, scaled for an in-sample 30% to 80% shift in share, as well as the explanatory power, number of observations, number of regressors, and the mean value of the dependent variable. Due to space constraints, we do not report estimated coefficients on the control variables as we do in other tables of estimates. We note that the vector of regressors, unless stated otherwise, follows Supplementary Table 5, capturing meteorological and road traffic conditions in the contemporaneous hour.

To illustrate, take as the dependent variable the 7-100 nm size range at 08:00, during the morning rush. From Supplementary Table 6 panels A to B to D (panel C is similar to panel B), the estimated coefficient on the gasoline share, scaled for a 30% to 80% shift, is a statistically significant: 3,559 cm^{-3} (s.e. 1,849 cm^{-3}) in the all-day-type sample; 5,805 cm^{-3} (s.e. 2,160 cm^{-3}) in the weekday-only sample; and 7,158 cm^{-3} (s.e. 2,112 cm^{-3}) in the weekday-only sample with wind direction control – see the rows marked "Hour: 8". In contrast, varying the time of day to 18:00 (but staying with the 7-100 nm size range), the estimate on the gasoline share is smaller and not significantly different from zero; see estimates of 1,251 cm^{-3} (s.e. 2,466 cm^{-3}) and 906 cm^{-3} (s.e. 2,298 cm^{-3}) in panels B and D, respectively, for the row marked "Hour: 18". Across the hours of the day in Supplementary Table 6, estimates are generally higher in the weekday-only sample relative to the all-day-type sample – panel B versus panel A. Moreover, estimates are higher during

the morning hours but otherwise similar when controlling for wind direction – panel D versus panel B.

If we fix the hour but vary the size range to 100-800 nm, the coefficient on the gasoline share is not significant; see estimates of -433 cm^{-3} (s.e. 794 cm^{-3}) and -697 cm^{-3} (s.e. 760 cm^{-3}) in Supplementary Table 7 panels B and D, respectively, for the row marked "Hour: 8". The examples illustrate that the empirical pattern reported in the main text is robust.

Supplementary Fig. 13 considers estimates from Supplementary Tables 6 and 7, panel D (weekday sample with wind direction control), and plots the 95% CI for the gasoline share coefficients over the diurnal cycle of the 7-100 nm and 100-800 nm size ranges. Similar to Figure 4a-b in the main text, for every hour of the day we plot a 95% CI for the gasoline share's association with the 7-100 nm size range (top panels), and another 95% CI for the gasoline share's association with the 100-800 nm size range (bottom panels). Here we show estimates not only in cm^{-3} (left panels) but also expressed as a proportion of the mean level in the sample for the given size range and hour (right panels). The 95% CI for the gasoline/7-100 nm association lies above zero between the hours of 07:00 and 11:00, but not in the afternoon hours. The point estimates are highest (and statistically significant) between 08:00 and 11:00. Such estimates amount to about 30% of the mean value of the dependent variable, particle levels in the 7-100 nm size range.

The bottom panels consider the 100-800 nm size range. The 95% CI for the gasoline share coefficients include zero over the entire diurnal cycle, in marked contrast to what we find for the 7-100 nm size range. The pattern is very similar to that reported in the main text.

Following Table 2 and Figure 4 in the main text, Supplementary Table 8 restricts the sample to the more seasonally homogeneous period from mid-summer to mid-fall 2011, and drops the quarter-of-year controls. We employ the specification in Supplementary Tables 6 and 7, panel D, weekday sample with wind direction controls. Panels A and B report on separate regressions of the 7-100 nm size range, excluding and including a linear time trend, respectively. Recall that the trend absorbs any monotonically (and linearly) varying unobserved determinants of the size distribution over this summer and fall period, rather than allowing these to potentially be confounded with the gasoline share. Similarly,

panels C and D consider the 100-800 nm size range as the dependent variable, not accounting and accounting for a trend, respectively.

Supplementary Fig. 14 plots the 95% CI for the gasoline/7-100 nm and gasoline/100-800 nm associations in the shorter summer/fall sample. We plot estimates from Supplementary Table 8, panels B and D, the specification that allows for a trend; however, allowing for a trend or not in this restricted sample makes little difference. Compared to Supplementary Fig. 13, the gasoline/7-100 nm association in Supplementary Fig. 14, estimated from the more seasonally homogeneous summer/fall sample, is more pronounced over the diurnal cycle; the gasoline/100-800 nm association becomes more negative but remains insignificant.

Estimates are robust to controlling for ridership on diesel buses in the metropolis' public transport system (Supplementary Fig. 6). Figure 3 in the main text shows the estimated gasoline/7-100 nm and gasoline/100-800nm associations at 08:00, during the morning rush, when the regression model controls for diesel bus ridership. Estimates are very similar to those reported in Figure 1 in the main text. Similarly, Supplementary Figs. 15 and 16, based on the full three-season and mid-summer to mid-fall samples respectively, demonstrate that the results presented in Supplementary Figs. 13 and 14 are robust to additionally controlling for the observed frequency of public transit diesel buses passing through the university campus at the time of sampling.

Supplementary Figure 13. Estimated changes in 7-100 nm and 100-800 nm particle levels over the diurnal cycle associated with a rise in blended gasoline (E20/E25) use in the flex-fuel light-vehicle fleet from 30 to 80 percentage points.

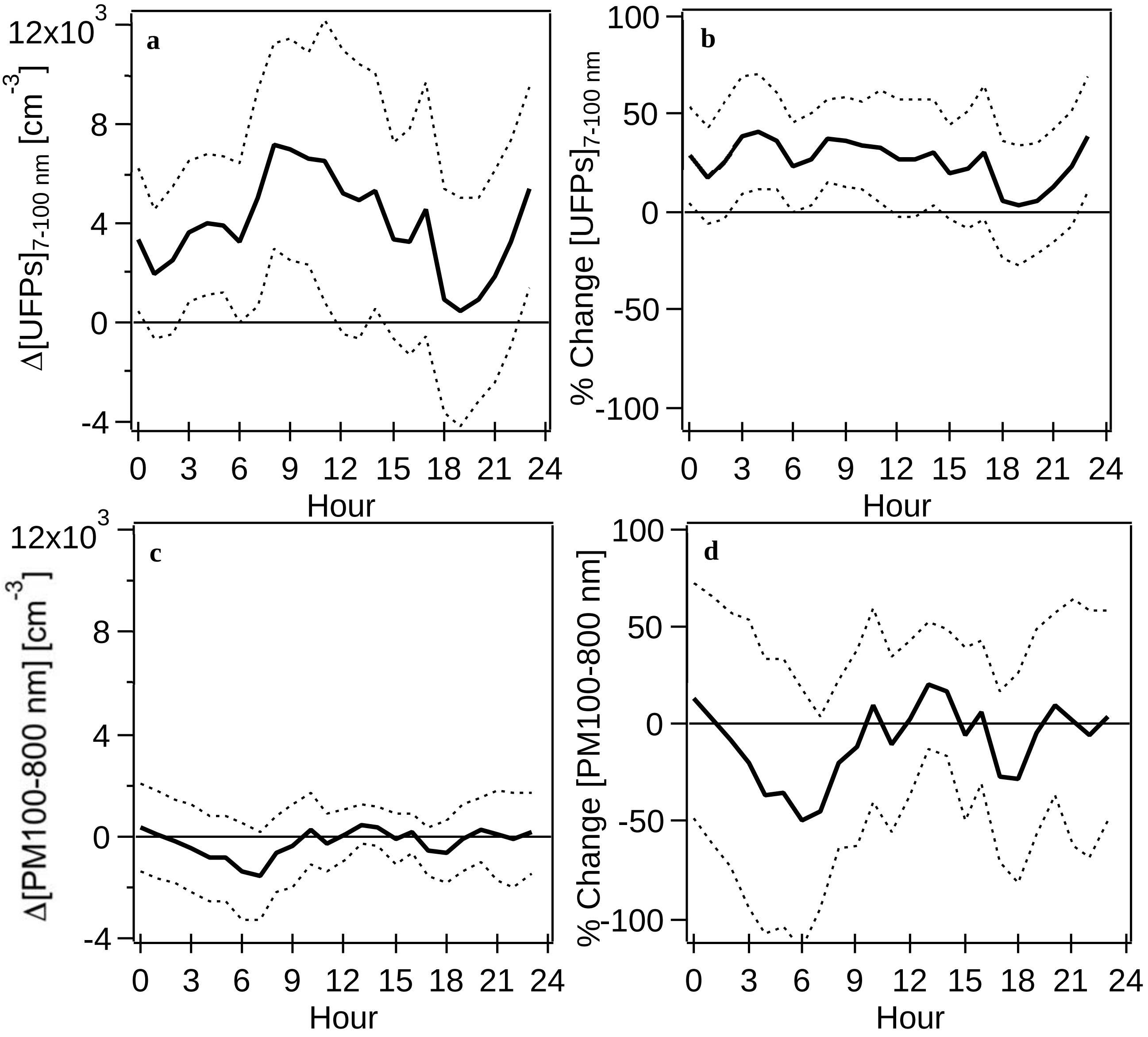

The panels report 95% CI for the 7-100 nm (**top**) and 100-800 nm (**bottom**) size ranges, from Supplementary Tables 6 and 7, respectively, panel D (weekday sample with wind direction control), both in cm^{-3} (**left**) and expressed as proportion of mean particle levels in the given size range and hour in the sample (**right**).

Supplementary Figure 14. Estimated changes in 7-100 nm and 100-800 nm particle levels when restricting the estimation sample to a more seasonally homogeneous mid-summer to mid-fall period.

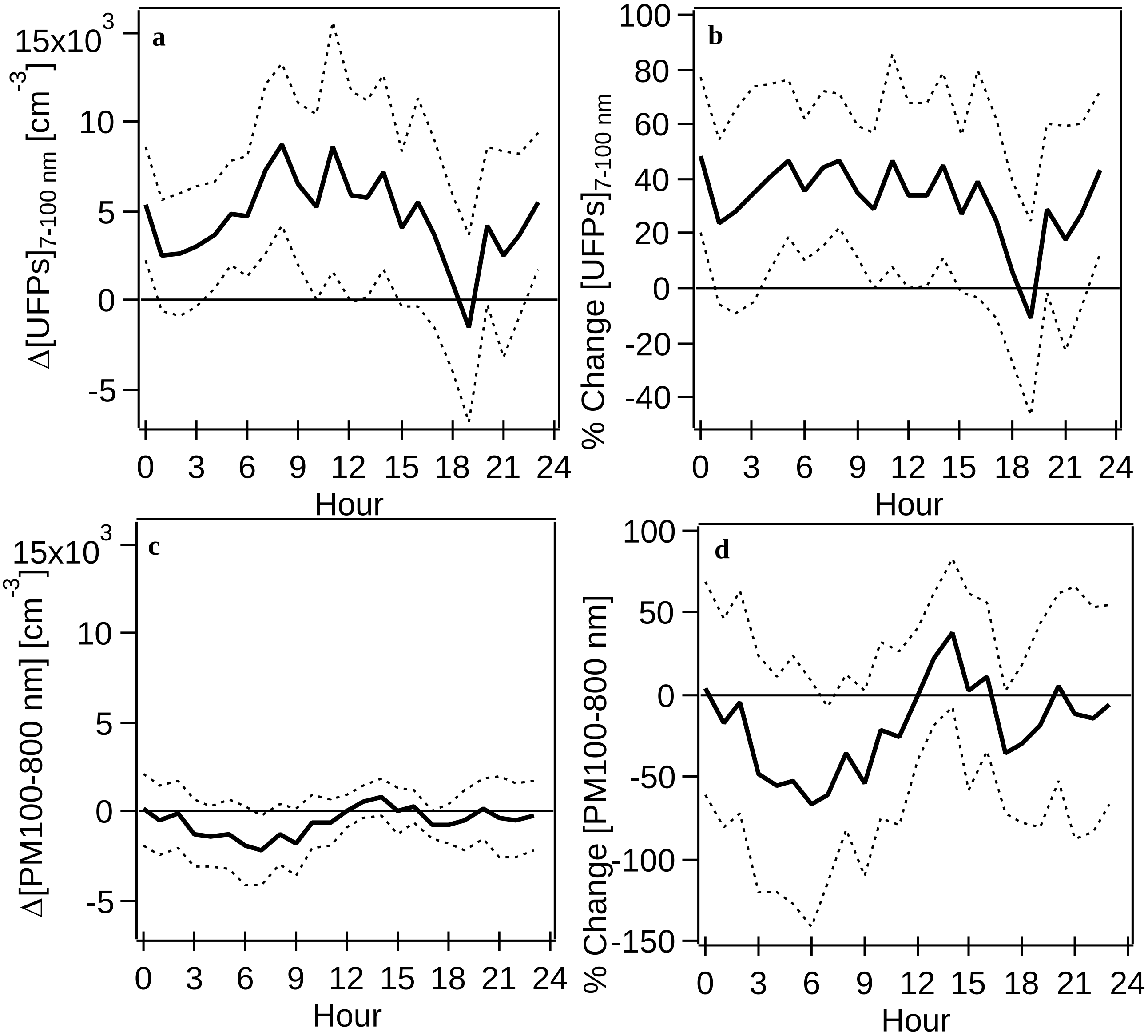

Variation in submicron particle levels associated with a rise in blended gasoline (E20/E25) use in the flex-fuel light-vehicle fleet from 30 to 80 percentage points. The panels report 95% CI for the 7-100 nm (**top**) and 100-800 nm (**bottom**) size ranges, from Supplementary Table 8, panels B and D, respectively, both in cm^{-3} (**left**) and expressed as proportion of mean particle levels in the given size range and hour in the sample (**right**).

Supplementary Figure 15. Sensitivity to diesel control for changes in submicron particle levels when using the full three-season sample of Supplementary Fig. 13.

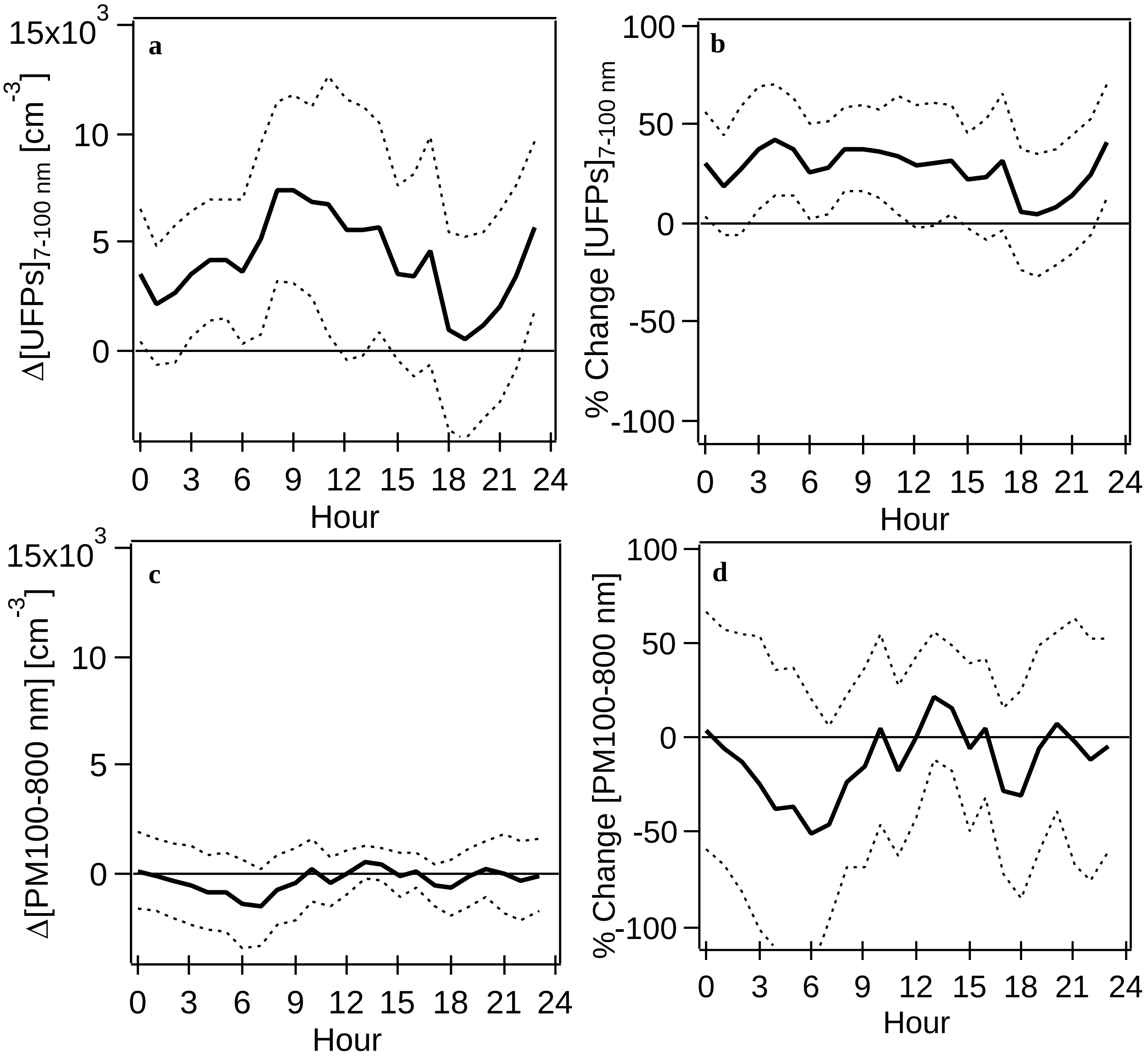

Variation in submicron particle levels associated with a rise in blended gasoline (E20/E25) use in the flex-fuel light-vehicle fleet from 30 to 80 percentage points. The panels follow the specification of Supplementary Fig. 13, reporting 95% CI for the 7-100 nm (**top**) and 100-800 nm (**bottom**) size ranges, both in cm^{-3} (**left**) and expressed as proportion of mean particle levels in the given size range and hour in the sample (**right**), but additionally control for the recorded frequency of public transit diesel buses passing through the university campus at the time of sampling.

Supplementary Figure 16. Sensitivity to diesel control for changes in submicron particle levels when restricting the estimation sample to the more seasonally homogeneous mid-summer to mid-fall period of Supplementary Fig. 14.

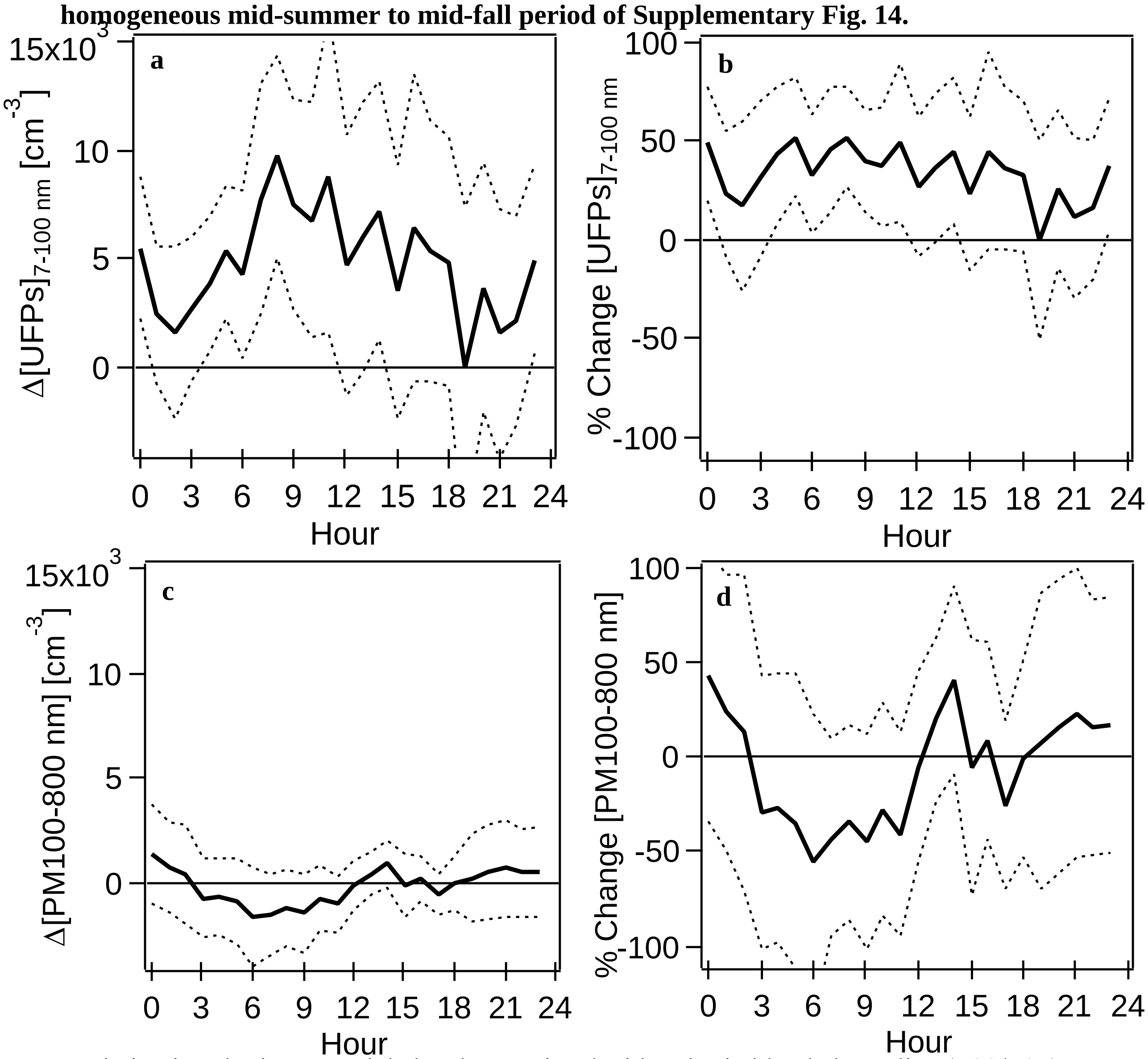

Variation in submicron particle levels associated with a rise in blended gasoline (E20/E25) use in the flex-fuel light-vehicle fleet from 30 to 80 percentage points. The panels follow the specification of Supplementary Fig. 14, reporting 95% CI for the 7-100 nm (**top**) and 100-800 nm (**bottom**) size ranges, both in cm^{-3} (**left**) and expressed as proportion of mean particle levels in the given size range and hour in the sample (**right**), but additionally control for the recorded frequency of public transit diesel buses passing through the university campus at the time of sampling.

Supplementary Table 6. Associations between the nanoparticle size distribution and the gasoline-ethanol fuel mix by hour of the day.

Dependent variable: **UFP 7-100 nm (cm^{-3})**

Key regressor of interest: **Share of Gasoline E20 over Ethanol E100 in the flex-fuel fleet increasing from 30 to 80%**

Control for unobserved seasonality: **Include quarter-of-year fixed effects (full period of field campaign)**

	Coefficient	Standard error	R^2	Number observ.	Number regress.	Mean of dep.var.	Coefficient	Standard error	R^2	Number observ.	Number regress.	Mean of dep.var.
Specification:	A. OLS + bootstrapping, all days of the week						B. OLS + bootstrapping, non-holiday weekdays only					
Hour: 0	3,688	(1,363)	56.1%	184	21	12,131	3,316	(1,491)	57.1%	117	16	11,704
Hour: 1	1,681	(1,216)	61.2%	184	20	11,140	2,591	(1,340)	60.6%	117	15	10,757
Hour: 2	1,596	(1,353)	55.3%	184	20	10,301	2,311	(1,383)	57.9%	117	15	9,860
Hour: 3	1,982	(1,389)	46.4%	188	20	9,599	3,173	(1,523)	50.9%	120	15	9,371
Hour: 4	1,569	(1,370)	47.2%	188	20	9,601	2,826	(1,522)	55.4%	120	14	9,744
Hour: 5	2,150	(1,426)	44.2%	188	20	10,491	3,232	(1,554)	46.2%	120	14	11,013
Hour: 6	2,444	(1,559)	46.4%	188	20	12,771	3,831	(1,752)	46.0%	120	14	13,927
Hour: 7	2,121	(2,028)	53.7%	188	22	16,593	4,569	(2,173)	53.9%	120	17	18,268
Hour: 8	3,559	(1,849)	57.7%	187	22	17,901	5,805	(2,160)	54.3%	120	16	19,570
Hour: 9	3,960	(2,152)	55.0%	187	22	18,012	5,653	(2,157)	57.3%	120	16	19,579
Hour: 10	2,072	(2,003)	43.7%	181	22	18,224	5,633	(2,230)	52.5%	115	16	19,306
Hour: 11	1,956	(2,633)	33.1%	181	22	18,803	5,733	(3,113)	40.9%	115	16	19,631
Hour: 12	3,192	(2,659)	31.3%	184	22	18,734	4,868	(3,052)	35.2%	117	16	19,241
Hour: 13	5,414	(2,521)	28.9%	187	22	17,584	5,461	(2,844)	26.1%	120	17	18,376
Hour: 14	3,587	(2,148)	30.8%	187	22	16,676	6,291	(2,429)	23.2%	120	17	17,642
Hour: 15	1,671	(1,837)	47.8%	183	22	15,329	3,403	(1,957)	46.7%	116	16	16,413
Hour: 16	1,680	(2,011)	46.4%	183	22	13,920	3,557	(2,256)	49.0%	116	16	15,152
Hour: 17	1,118	(2,004)	52.5%	182	22	13,578	3,814	(2,489)	49.1%	116	16	14,940
Hour: 18	1,867	(1,995)	53.8%	183	23	13,563	1,251	(2,466)	49.9%	116	16	14,933
Hour: 19	-486	(1,952)	45.1%	182	23	13,751	47	(2,164)	41.6%	116	17	14,720
Hour: 20	1,205	(1,951)	51.9%	183	23	13,939	1,139	(2,147)	52.6%	117	16	14,528
Hour: 21	3,288	(1,584)	55.1%	182	23	13,941	2,429	(1,908)	57.2%	116	18	14,510
Hour: 22	3,086	(1,552)	53.1%	184	22	13,676	3,204	(1,977)	49.9%	118	17	14,284
Hour: 23	4,620	(1,616)	54.9%	183	21	13,291	4,215	(1,980)	53.9%	117	16	13,759
Specification:	C. 2SLS, non-holiday weekdays only						D. OLS + bootstrapping, weekdays, control wind direction					
Hour: 0	3,344	(1,352)	57.1%	117	16	11,704	3,352	(1,492)	61.9%	117	20	11,704
Hour: 1	2,575	(1,269)	60.6%	117	15	10,757	1,993	(1,334)	67.4%	116	19	10,820
Hour: 2	2,325	(1,210)	57.9%	117	15	9,860	2,523	(1,512)	63.1%	116	19	9,828
Hour: 3	3,165	(1,493)	50.9%	120	15	9,371	3,669	(1,450)	59.3%	120	19	9,371
Hour: 4	2,777	(1,391)	55.4%	120	15	9,744	3,962	(1,462)	64.5%	120	18	9,744
Hour: 5	3,213	(1,463)	46.2%	120	15	11,013	3,925	(1,398)	57.9%	120	18	11,013
Hour: 6	3,801	(1,737)	46.0%	120	15	13,927	3,222	(1,642)	58.8%	120	18	13,927
Hour: 7	4,520	(2,205)	53.9%	120	17	18,268	5,015	(2,236)	59.7%	120	21	18,268
Hour: 8	5,780	(1,997)	54.3%	120	16	19,570	7,158	(2,112)	60.7%	120	20	19,570
Hour: 9	5,636	(2,081)	57.3%	120	16	19,579	6,995	(2,301)	66.3%	120	20	19,579
Hour: 10	5,562	(2,107)	52.5%	115	16	19,306	6,594	(2,181)	60.1%	115	19	19,306
Hour: 11	5,663	(2,789)	40.9%	115	17	19,631	6,551	(2,907)	44.4%	115	19	19,631
Hour: 12	4,828	(2,644)	35.2%	117	17	19,241	5,225	(2,928)	37.4%	117	19	19,241
Hour: 13	5,346	(2,259)	26.1%	120	17	18,376	4,890	(2,817)	31.5%	118	20	18,320
Hour: 14	6,204	(1,942)	23.2%	120	17	17,642	5,318	(2,428)	28.1%	118	20	17,574
Hour: 15	3,406	(1,839)	46.7%	116	17	16,413	3,337	(2,016)	49.7%	114	19	16,338
Hour: 16	3,537	(2,111)	49.0%	116	17	15,152	3,264	(2,342)	55.2%	115	19	15,151
Hour: 17	3,779	(2,220)	49.1%	116	17	14,940	4,564	(2,613)	53.1%	114	19	14,968
Hour: 18	1,186	(2,320)	49.9%	116	17	14,933	906	(2,298)	56.5%	114	19	14,938
Hour: 19	29	(2,074)	41.6%	116	17	14,720	416	(2,353)	41.9%	113	21	14,828
Hour: 20	1,142	(1,993)	52.6%	117	18	14,528	934	(2,105)	54.8%	115	20	14,622
Hour: 21	2,466	(1,791)	57.2%	116	18	14,510	1,867	(2,173)	59.9%	114	22	14,575
Hour: 22	3,282	(1,781)	49.9%	118	17	14,284	3,227	(2,126)	52.2%	117	20	14,316
Hour: 23	4,271	(1,656)	53.9%	117	16	13,759	5,416	(2,067)	60.7%	117	20	13,759

Notes: Each row by panel corresponds to a separate regression estimated on a sample that is restricted to a specific hour of the day. Samples exclude the colder months of June to September, and may include all days of the week or only non-holiday weekdays as indicated. Panels A, B and D report Ordinary Least Squares estimates, with standard errors calculated by bootstrapping, as in the odd-numbered columns of Supplementary Table 5. Panel C reports Two-Stage Least Squares estimates, as in the even-numbered columns of Supplementary Table 5. Panel A considers all types of day, whereas panels B to D restrict the sample to non-holiday weekdays only. Panel D further controls for wind direction. See notes to Supplementary Table 5. For brevity, only the estimates on the gasoline share are shown (not the estimates on control variables).

Supplementary Table 7. Associations between the submicron particle size distribution and the gasoline-ethanol fuel mix by hour of the day.

Dependent variable: **PM 100-800 nm (cm^{-3})**

Key regressor of interest: **Share of Gasoline E20 over Ethanol E100 in the flex-fuel fleet increasing from 30 to 80%**

Control for unobserved seasonality: **Include quarter-of-year fixed effects (full period of field campaign)**

	Coefficient	Standard error	R^2	Number observ.	Number regress.	Mean of dep.var.	Coefficient	Standard error	R^2	Number observ.	Number regress.	Mean of dep.var.
Specification:	A. OLS + bootstrapping, all days of the week						B. OLS + bootstrapping, non-holiday weekdays only					
Hour: 0	160	(858)	61.8%	184	21	2,840	232	(902)	67.1%	117	16	2,797
Hour: 1	52	(825)	63.7%	184	20	2,737	493	(841)	69.0%	117	15	2,651
Hour: 2	117	(849)	63.6%	184	20	2,594	99	(771)	73.1%	117	15	2,518
Hour: 3	58	(817)	55.4%	188	20	2,409	-212	(940)	60.0%	120	15	2,350
Hour: 4	-343	(752)	54.4%	188	20	2,351	-1,000	(856)	65.5%	120	14	2,339
Hour: 5	-225	(742)	50.8%	188	20	2,386	-1,215	(821)	57.6%	120	14	2,413
Hour: 6	-90	(867)	49.5%	188	20	2,700	-1,473	(982)	57.6%	120	14	2,833
Hour: 7	-183	(871)	51.1%	188	22	3,162	-1,061	(918)	58.5%	120	17	3,454
Hour: 8	2	(760)	55.6%	187	22	3,118	-433	(794)	59.8%	120	16	3,426
Hour: 9	-6	(728)	57.6%	187	22	2,882	-259	(788)	57.8%	120	16	3,187
Hour: 10	-250	(654)	47.6%	181	22	2,562	-141	(792)	49.5%	115	16	2,800
Hour: 11	-399	(473)	45.9%	181	22	2,327	-327	(579)	47.5%	115	16	2,497
Hour: 12	290	(387)	44.2%	184	22	2,259	257	(465)	52.7%	117	16	2,372
Hour: 13	624	(343)	46.4%	187	22	2,209	571	(395)	51.5%	120	17	2,304
Hour: 14	412	(375)	43.1%	187	22	2,267	497	(431)	50.4%	120	17	2,324
Hour: 15	22	(375)	40.0%	183	22	2,202	170	(449)	49.0%	116	16	2,260
Hour: 16	-41	(364)	40.1%	183	22	2,024	175	(407)	50.4%	116	16	2,101
Hour: 17	-278	(481)	39.9%	182	22	2,051	-380	(505)	45.2%	116	16	2,156
Hour: 18	-274	(482)	40.4%	183	23	2,149	-453	(572)	42.0%	116	16	2,292
Hour: 19	-375	(485)	51.2%	182	23	2,304	-360	(558)	55.6%	116	17	2,472
Hour: 20	398	(566)	65.5%	183	23	2,539	225	(690)	67.4%	117	16	2,708
Hour: 21	478	(663)	63.0%	182	23	2,666	456	(797)	63.6%	116	18	2,789
Hour: 22	9	(669)	62.7%	184	22	2,717	-38	(873)	60.9%	118	17	2,841
Hour: 23	29	(708)	63.6%	183	21	2,816	143	(780)	65.2%	117	16	2,970
Specification:	C. 2SLS, non-holiday weekdays only						D. OLS + bootstrapping, weekdays, control wind direction					
Hour: 0	212	(829)	67.1%	117	16	2,797	353	(866)	75.3%	117	20	2,797
Hour: 1	454	(782)	69.0%	117	15	2,651	67	(868)	74.3%	116	19	2,673
Hour: 2	74	(686)	73.1%	117	15	2,518	-197	(841)	76.6%	116	19	2,528
Hour: 3	-226	(861)	60.0%	120	15	2,350	-468	(884)	65.7%	120	19	2,350
Hour: 4	-1,014	(808)	65.5%	120	15	2,339	-871	(845)	68.9%	120	18	2,339
Hour: 5	-1,223	(779)	57.6%	120	15	2,413	-849	(849)	61.2%	120	18	2,413
Hour: 6	-1,483	(894)	57.6%	120	15	2,833	-1,381	(958)	65.5%	120	18	2,833
Hour: 7	-1,073	(873)	58.5%	120	17	3,454	-1,556	(864)	70.7%	120	21	3,454
Hour: 8	-428	(768)	59.8%	120	16	3,426	-697	(760)	66.7%	120	20	3,426
Hour: 9	-267	(746)	57.8%	120	16	3,187	-379	(815)	63.2%	120	20	3,187
Hour: 10	-140	(699)	49.5%	115	16	2,800	281	(716)	57.1%	115	19	2,800
Hour: 11	-323	(511)	47.5%	115	17	2,497	-254	(574)	49.4%	115	19	2,497
Hour: 12	250	(437)	52.7%	117	17	2,372	70	(486)	55.2%	117	19	2,372
Hour: 13	556	(400)	51.5%	120	17	2,304	457	(383)	54.7%	118	20	2,309
Hour: 14	493	(364)	50.4%	120	17	2,324	384	(387)	55.9%	118	20	2,314
Hour: 15	171	(413)	49.0%	116	17	2,260	-104	(501)	53.6%	114	19	2,231
Hour: 16	165	(387)	50.4%	116	17	2,101	135	(394)	54.1%	115	19	2,109
Hour: 17	-407	(491)	45.2%	116	17	2,156	-584	(483)	59.7%	114	19	2,166
Hour: 18	-463	(561)	42.0%	116	17	2,292	-635	(627)	49.0%	114	19	2,294
Hour: 19	-380	(519)	55.6%	116	17	2,472	-107	(669)	60.1%	113	21	2,479
Hour: 20	215	(677)	67.4%	117	18	2,708	272	(652)	70.6%	115	20	2,724
Hour: 21	427	(726)	63.6%	116	18	2,789	31	(914)	69.6%	114	22	2,803
Hour: 22	-56	(853)	60.9%	118	17	2,841	-146	(934)	62.2%	117	20	2,856
Hour: 23	122	(764)	65.2%	117	16	2,970	115	(823)	67.1%	117	20	2,970

Notes: Each row by panel corresponds to a separate regression estimated on a sample that is restricted to a specific hour of the day. Samples exclude the colder months of June to September, and may include all days of the week or only non-holiday weekdays as indicated. Panels A, B and D report Ordinary Least Squares estimates, with standard errors calculated by bootstrapping, as in the odd-numbered columns of Supplementary Table 5. Panel C reports Two-Stage Least Squares estimates, as in the even-numbered columns of Supplementary Table 5. Panel A considers all types of day, whereas panels B to D restrict the sample to non-holiday weekdays only. Panel D further controls for wind direction. See notes to Supplementary Table 5. For brevity, only the estimates on the gasoline share are shown (not the estimates on control variables).

Supplementary Table 8. Associations between the submicron particle size distribution and the gasoline-ethanol fuel mix by hour of the day.

Dependent variable: **PM 7-100 nm (cm^{-3})** in the top panels**,** or **PM 100-800 nm (cm^{-3})** in the bottom panels

Key regressor of interest: **Share of Gasoline E20 over Ethanol E100 in the flex-fuel fleet increasing from 30 to 80%**

Control for unobserved seasonality: **Restrict sample to summer/fall months of January to May 2011**, and add trend in the right panels

	Coefficient	Standard error	R^2	Number observ.	Number regress.	Mean of dep.var.	Coefficient	Standard error	R^2	Number observ.	Number regress.	Mean of dep.var.
Specification:	A. 7-100nm size range (cm^{-3}), no trend						B. 7-100nm size range (cm^{-3}), add linear trend					
Hour: 0	4,891	(1,491)	71.8%	77	16	11,158	5,366	(1,625)	72.3%	77	17	11,158
Hour: 1	2,652	(1,537)	75.5%	77	17	10,270	2,460	(1,584)	75.7%	77	18	10,270
Hour: 2	2,553	(1,647)	70.2%	76	17	9,197	2,582	(1,745)	70.2%	76	18	9,197
Hour: 3	3,037	(1,663)	61.8%	80	16	8,606	3,003	(1,733)	61.8%	80	17	8,606
Hour: 4	3,604	(1,464)	63.6%	80	16	8,949	3,662	(1,551)	63.6%	80	17	8,949
Hour: 5	4,606	(1,516)	62.5%	80	15	10,232	4,849	(1,482)	62.7%	80	16	10,232
Hour: 6	4,463	(1,691)	60.4%	80	16	12,884	4,674	(1,717)	60.5%	80	17	12,884
Hour: 7	7,021	(2,324)	58.0%	80	19	16,789	7,342	(2,424)	58.2%	80	20	16,789
Hour: 8	8,168	(2,109)	69.4%	80	18	18,659	8,713	(2,326)	69.8%	80	19	18,659
Hour: 9	5,933	(1,983)	74.1%	80	18	18,585	6,515	(2,288)	74.3%	80	19	18,585
Hour: 10	6,515	(2,382)	67.6%	76	17	18,205	5,225	(2,659)	68.5%	76	18	18,205
Hour: 11	8,536	(3,084)	45.0%	76	17	18,002	8,545	(3,584)	45.0%	76	18	18,002
Hour: 12	5,773	(3,003)	40.1%	76	17	17,347	5,830	(3,009)	40.1%	76	18	17,347
Hour: 13	5,378	(2,347)	28.1%	76	18	16,551	5,701	(2,830)	28.3%	76	19	16,551
Hour: 14	6,394	(2,350)	27.0%	77	18	16,085	7,201	(2,777)	27.8%	77	19	16,085
Hour: 15	2,895	(1,798)	42.1%	74	17	15,001	4,031	(2,224)	43.7%	74	18	15,001
Hour: 16	4,403	(2,639)	43.3%	73	16	14,134	5,468	(3,008)	44.9%	73	17	14,134
Hour: 17	2,834	(2,666)	55.0%	73	17	14,599	3,719	(2,684)	57.1%	73	18	14,599
Hour: 18	1,006	(2,529)	65.6%	73	16	14,838	951	(2,504)	66.6%	73	17	14,838
Hour: 19	-940	(2,702)	61.8%	72	19	14,712	-1,580	(2,695)	64.8%	72	20	14,712
Hour: 20	3,701	(2,272)	62.2%	74	18	14,267	4,164	(2,266)	64.2%	74	19	14,267
Hour: 21	2,054	(2,723)	56.7%	73	20	13,947	2,544	(2,929)	57.1%	73	21	13,947
Hour: 22	2,888	(2,192)	52.0%	77	18	13,621	3,645	(2,345)	53.1%	77	19	13,621
Hour: 23	4,528	(1,844)	57.9%	77	18	12,964	5,528	(1,960)	59.8%	77	19	12,964
Specification:	C. 100-800nm size range (cm^{-3}), no trend						D. 100-800nm size range (cm^{-3}), add linear trend					
Hour: 0	838	(834)	79.7%	77	16	3,092	124	(1,019)	81.9%	77	17	3,092
Hour: 1	536	(953)	74.8%	77	17	2,952	-509	(965)	82.2%	77	18	2,952
Hour: 2	816	(855)	79.0%	76	17	2,818	-129	(968)	83.0%	76	18	2,818
Hour: 3	-492	(943)	69.6%	80	16	2,594	-1,249	(958)	73.6%	80	17	2,594
Hour: 4	-644	(893)	71.6%	80	16	2,565	-1,401	(859)	75.6%	80	17	2,565
Hour: 5	-545	(941)	64.3%	80	15	2,576	-1,331	(988)	68.9%	80	16	2,576
Hour: 6	-1,060	(939)	65.2%	80	16	2,962	-1,978	(1,130)	69.4%	80	17	2,962
Hour: 7	-1,312	(1,019)	72.3%	80	19	3,584	-2,192	(978)	76.9%	80	20	3,584
Hour: 8	-751	(798)	74.3%	80	18	3,577	-1,249	(852)	76.0%	80	19	3,577
Hour: 9	-662	(808)	65.2%	80	18	3,255	-1,749	(942)	71.9%	80	19	3,255
Hour: 10	41	(679)	64.1%	76	17	2,762	-595	(755)	66.7%	76	18	2,762
Hour: 11	-485	(585)	54.3%	76	17	2,485	-644	(667)	54.6%	76	18	2,485
Hour: 12	-23	(453)	64.6%	76	17	2,315	10	(483)	64.7%	76	18	2,315
Hour: 13	449	(482)	58.7%	76	18	2,251	500	(461)	58.8%	76	19	2,251
Hour: 14	767	(474)	57.6%	77	18	2,250	846	(526)	57.7%	77	19	2,250
Hour: 15	474	(573)	48.2%	74	17	2,224	40	(683)	51.2%	74	18	2,224
Hour: 16	551	(460)	45.8%	73	16	2,061	230	(471)	48.8%	73	17	2,061
Hour: 17	-361	(496)	64.1%	73	17	2,196	-783	(419)	72.5%	73	18	2,196
Hour: 18	-678	(628)	62.8%	73	16	2,368	-716	(579)	70.0%	73	17	2,368
Hour: 19	-702	(697)	70.6%	72	19	2,673	-503	(850)	73.1%	72	20	2,673
Hour: 20	192	(933)	74.5%	74	18	2,927	148	(847)	74.6%	74	19	2,927
Hour: 21	311	(975)	73.6%	73	20	2,986	-329	(1,173)	75.8%	73	21	2,986
Hour: 22	297	(1,057)	68.3%	77	18	3,064	-470	(1,076)	70.9%	77	19	3,064
Hour: 23	705	(938)	70.3%	77	18	3,172	-201	(987)	73.6%	77	19	3,172

Notes: Each row by panel corresponds to a separate regression estimated on a sample that is restricted to a specific hour of the day. As in Supplementary Table 6 panel D, all samples include non-holiday weekdays only, and all specifications include controls for wind direction. Ordinary Least Squares estimates, with standard errors calculated by bootstrapping, as in the odd-numbered columns of Supplementary Table 5. See notes to Supplementary Table 5. For brevity, only the estimates on the gasoline share are shown (not the estimates on control variables). All specifications exclude quarter-of-year fixed effects, and panels B and D include a linear trend.

Supplementary Note 6. Graphical illustration of residuals. Supplementary Fig. 17 provides an intuitive illustration of the penetration of gasoline and the differential result for the 7-100 nm and 100-800 nm size ranges. We base this illustration on estimates from Figures 2 and 4 in the main text (also in Supplementary Table 8, panels B and D), namely regressions by hour, restricting the sample to non-holiday weekdays between January 20 and May 31, 2011, and including wind direction and a linear trend in the vector of controls – all with a view to limiting unobserved determinants of the particle size distribution, which might confound the association with the fuel mix.

Panel a shows the variation in the gasoline share in the flex-fuel vehicle fleet, $\hat{s}_t^{\mathrm{gas}}$, over the dates in this seasonally homogeneous sample. We only show dates for which we have DMPS measurements. Panels b and c plot the evolution of raw data measured at 08:00 for the 7-100 nm and 100-800 nm size ranges, respectively, over this same period. Even in this more seasonally homogeneous sample – notice that there is no obvious trend – and fixing the hour of the reading (08:00, one-hour average), there is substantial variability in the raw data, much as there is variability in the raw 24-hour PM2.5 data (Supplementary Fig. 3). For example, the 7-100 nm values range between under 10,000 cm^{-3} to almost 40,000 cm^{-3}. We then "subtract" (i.e., "partial out") from each series of values – the 7-100 nm values at 08:00, the 100-800 nm values at 08:00, and $\hat{s}_t^{\mathrm{gas}}$ – any co-variation with observed meteorological, atmospheric, and road traffic conditions, as well as systematic day-of-the-week (e.g., Monday versus Friday) and trending variation. The corrected co-variation is shown in the scatterplot of residuals: 7-100 nm against the gasoline share in panel d, and 100-800 nm against the gasoline share in panel e. The best linear predictors are also shown. The strong positive association is evident in panel d. (The temporal evolution of these residuals, day by day, is plotted in Figure 2d in the main text.) There is no obvious association in panel e (100-800 nm at 08:00). We repeat the exercise for 7-100 nm and 100-800 nm measurements at 18:00 in panels f and g, where again there is no obvious relationship.

Supplementary Figure 17. Analysis of residuals for gasoline's association with the 7-100 nm and 100-800 nm size ranges at 08:00 and 18:00 on weekdays in the mid-summer to mid-fall 2011 sample.

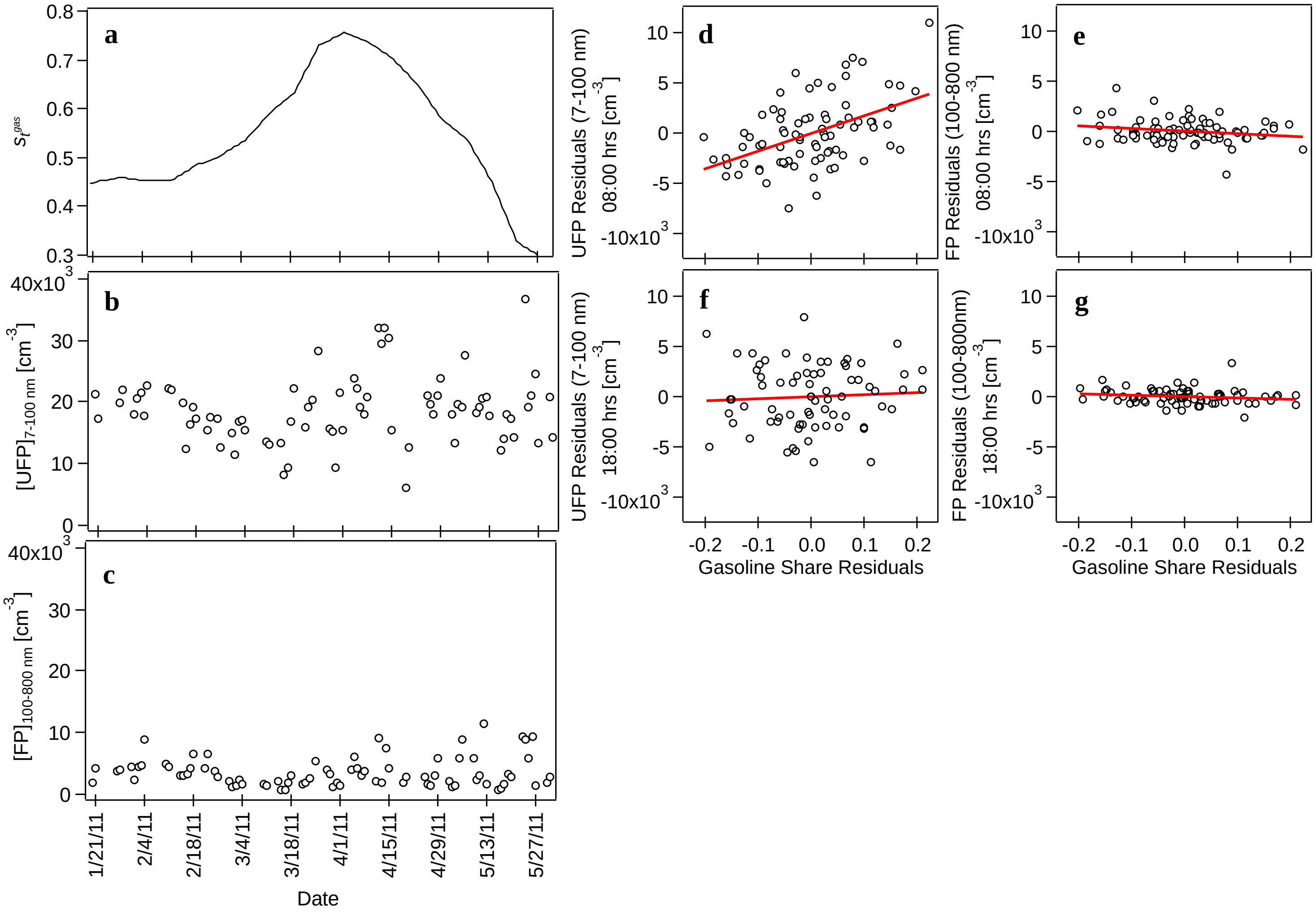

(**a**) Variation in the gasoline share in the flex-fuel light-vehicle fleet. (**b**) Raw data for the 7-100 nm size range measured at 08:00. (**c**) Raw data for the 100-800 nm size range measured at 08:00. (**d**) Residuals of 7-100 nm at 08:00 against gasoline share residuals. (**e**) Residuals of 100-800 nm at 08:00 against gasoline share residuals. (**f**) Residuals of 7-100 nm at 18:00 against gasoline share residuals. (**g**) Residuals of 100-800 nm at 18:00 against gasoline share residuals. Residuals based on the specification in Supplementary Table 8, panels B and D. The red line marks the best linear predictor.

Supplementary References

1 Salvo, A. & Huse, C. Build it, but will they come? Evidence from consumer choice between gasoline and sugarcane ethanol. *J Environ Econ Manag* **66**, 251-279, doi:10.1016/j.jeem.2013.04.001 (2013).

2 EPA. in *The National Ambient Air Quality Standards for Particle Pollution* (US Environmental Protection Agency, Research Triangle Park, 2012).

3 Salvo, A. & Geiger, F. M. Reduction in local ozone levels in urban São Paulo due to a shift from ethanol to gasoline use. *Nature Geoscience* **7**, 450-458, doi:10.1038/ngeo2144 (2014).
